# High-Fat Diet-Induced Excessive Accumulation of Cerebral Cholesterol Esters and Microglial Dysfunction Exacerbate Alzheimer's Disease Pathology in *APP*^*NL−G−F*^ mice

**DOI:** 10.1007/s12035-025-05052-8

**Published:** 2025-05-17

**Authors:** Shuhan Yang, Hirofumi Miyazaki, Tunyanat Wannakul, Eiko Amo, Takaomi Saido, Takashi Saito, Hiroki Sasaguri, Motoko Maekawa, Yuji Owada

**Affiliations:** 1 Department of Organ Anatomy, Graduate School of Medicine, Tohoku University, Sendai, Miyagi 980-8575 Japan; 2 Faculty of Medicine, Khon Kaen University, Mueang Khon Kaen District, Khon Kaen, 40002 Thailand; 3 Laboratory for Proteolytic Neuroscience, RIKEN Center for Brain Science, Wako, Saitama 351-0198 Japan; 4 Department of Neurocognitive Science, Institute of Brain Science, Nagoya City University Graduate School of Medical Sciences, Nagoya, Aichi 467-8601 Japan; 5 Dementia Pathophysiology Collaboration Unit, RIKEN Center for Brain Science, Wako, Saitama 351-0198 Japan; 6 Fukushima Institute for Research, Education and Innovation, Namie, Fukushima 979-1521 Japan

**Keywords:** Alzheimer’s disease, High-fat diet, Microglial function, Lipid metabolism, Lipid droplet

## Abstract

Epidemiological studies have identified high-fat diet (HFD)-induced obesity as a risk factor for Alzheimer's disease (AD), yet the underlying molecular mechanisms remain inadequately elucidated. Microglia, the brain’s innate immune cells, are pivotal in AD brain by engulfing β-amyloid (Aβ) peptides and compacting poorly consolidated Aβ plaques. Microglia are highly susceptible to the metabolic milieu; however, it is unclear how long-term HFD alters the lipid environment and influences microglial phenotype in AD brains. In this study, *APP*^*NL−G−F*^ knock-in AD model mice were fed an HFD for 9–27 weeks and subsequently analyzed for Aβ pathology and microglial function. Our findings indicated that HFD intake accelerated Aβ deposition, attenuated the recruitment of microglia to the plaques and impaired their phagocytic activity, while also promoting the accumulation of intracellular lipid droplets (LDs). Lipidomic analyses revealed that HFD, in synergy with AD pathology, increased the proportion of cholesterol esters in the cerebral cortex. In vitro, oleic acid—a major HFD constituent—similarly diminished the phagocytic capacity of MG6 microglia and induced LDs accumulation, along with downregulation of gene sets of cholesterol efflux, phagocytosis and engulfment. Overall, these findings implied that HFD-induced perturbation in brain cholesterol homeostasis may compromise microglial activation and expedite AD progression in *APP*^*NL−G−F*^ mice.

**Supplementary Information:**

The online version contains supplementary material available at 10.1007/s12035-025-05052-8.

## Introduction

Alzheimer's disease (AD) is the most prevalent neurodegenerative disease among the elderly, characterized by the accumulation of β-amyloid (Aβ) plaques, neurofibrillary tangles, as well as memory, cognitive, language, and motor deficits. In the late stages, severe brain atrophy can lead to dysphagia, resulting in malnutrition and multi-organ failure [1]. Although the precise etiology remains unclear, human genetic studies indicate that the majority of AD cases are sporadic, arising from gene-environment interactions [2].

A meta-analysis based on extensive clinical data indicates that obesity significantly increases the risk of AD independently of factors such as diabetes and the APOE4 allele mutation [3]. Additionally, studies employing animal models revealed the impact of high-fat diet (HFD) on the development of AD. HFD might accelerate AD pathological deterioration through aggravating neuroinflammation in the hypothalamus and hippocampus [4, 5], upregulating the expression of amyloid precursor protein (*App*) and presenilin 2 (*Psen2*) genes [6], enhancing the cleavage of APP [7], and hindering Aβ degradation [8]. Some studies found that although HFD impaired cognition and memory in AD mouse models, it had no significant effect on the histopathological progression [9–11]. In contrast, other studies suggested that HFD alleviated AD pathology by improving blood–brain barrier integrity [12], reducing the leakage of fibrinogen from blood vessels into the brain parenchyma [13], and increasing apolipoprotein A-I (APOA-I) level [14]. Due to certain transgenic models exhibiting APP overexpression and other genetic backgrounds inconsistent with physiological conditions [15], studies on the impact of HFD on AD pathological progression have yet to yield consistent conclusions. Furthermore, there remains a paucity of research exploring how HFD ingestion perturbs the lipid milieu in AD brain and investigating the phenotype and function of glial cells, which are paramount to AD pathology.

Microglia, the resident immune cells of the central nervous system (CNS), play crucial roles under both physiological and pathological conditions. In AD brains, locally resident microglia are activated by the stimulation of excessive accumulated Aβ plaques, extend their processes, and then migrate toward the plaques, continuously internalizing Aβs [16]. In addition to phagocytosis, microglia act as barriers to restrict the radial expansion of Aβ plaques by encapsulating and compacting them, thereby mitigating their neurotoxic effects [17]. Functional activation of microglia is accompanied by changes in gene expression. Single-cell RNA sequencing has identified a subset of responsive microglia near Aβ plaques, exhibiting robust phagocytic activity and significant upregulation of genes related to lipid transport (*Trem2*, *Apoe*, and *Lpl*), suggesting a correlation between microglial activation and lipid metabolism [18–20]. Microglia express all genes required for glycolysis and oxidative phosphorylation, enabling them to adapt to various environmental stimuli and cellular stresses through metabolic switching [21, 22]. Conversely, metabolic interventions can also influence microglial function and phenotype [23]. Despite the identification of metabolic changes in microglia within AD brains, the impact of long-term HFD intake on microglial metabolism and function in pathological environments remains unexplored. Here, we hypothesize that HFD feeding may lead to microglial lipid metabolism failure by elevating lipid content in AD brains, thereby affecting microglial function and AD pathology.

This study utilized *APP*^*NL−G−F*^ AD model mice, which incorporate Swedish (KM670/671 NL) and Beyreuther/Iberian (I716 F) mutations into the endogenous *App* gene [24], thereby avoiding the overexpression issues seen in traditional transgenic mice, accurately reflecting the formation and distribution of Aβ plaques characteristic of human AD pathology. The mice were fed with a 60% fat HFD to study its effects on AD pathology and microglial function, with lower sucrose compared to a 45% fat HFD to minimize cardiovascular disease interference from sugar intake. Our results showed that HFD accelerated Aβ plaque deposition in *APP*^*NL−G−F*^ mice brain and worsened their spatial memory. These effects may be linked to the HFD-induced accumulation of excessive cholesterol esters (ChEs) in brain tissue, which led to lipid droplet (LD) overload in microglia and subsequently impaired their migration and phagocytic activity. In vitro experiments further confirmed that oleic acid (OA), the most abundant fatty acid in HFD, also caused ChE and LD overload in MG6 microglia, downregulated microglial activity in phagocytizing Aβ peptides and triggered a decrease in the expression of a series of genes related to cholesterol metabolism and phagocytosis. Taken together, this study highlights the underappreciated role of HFD-induced lipid metabolic dysregulation in microglial function and AD progression.

## Materials and Methods

### Animals and HFD-induced Obesity Model

**Fig. 1:**
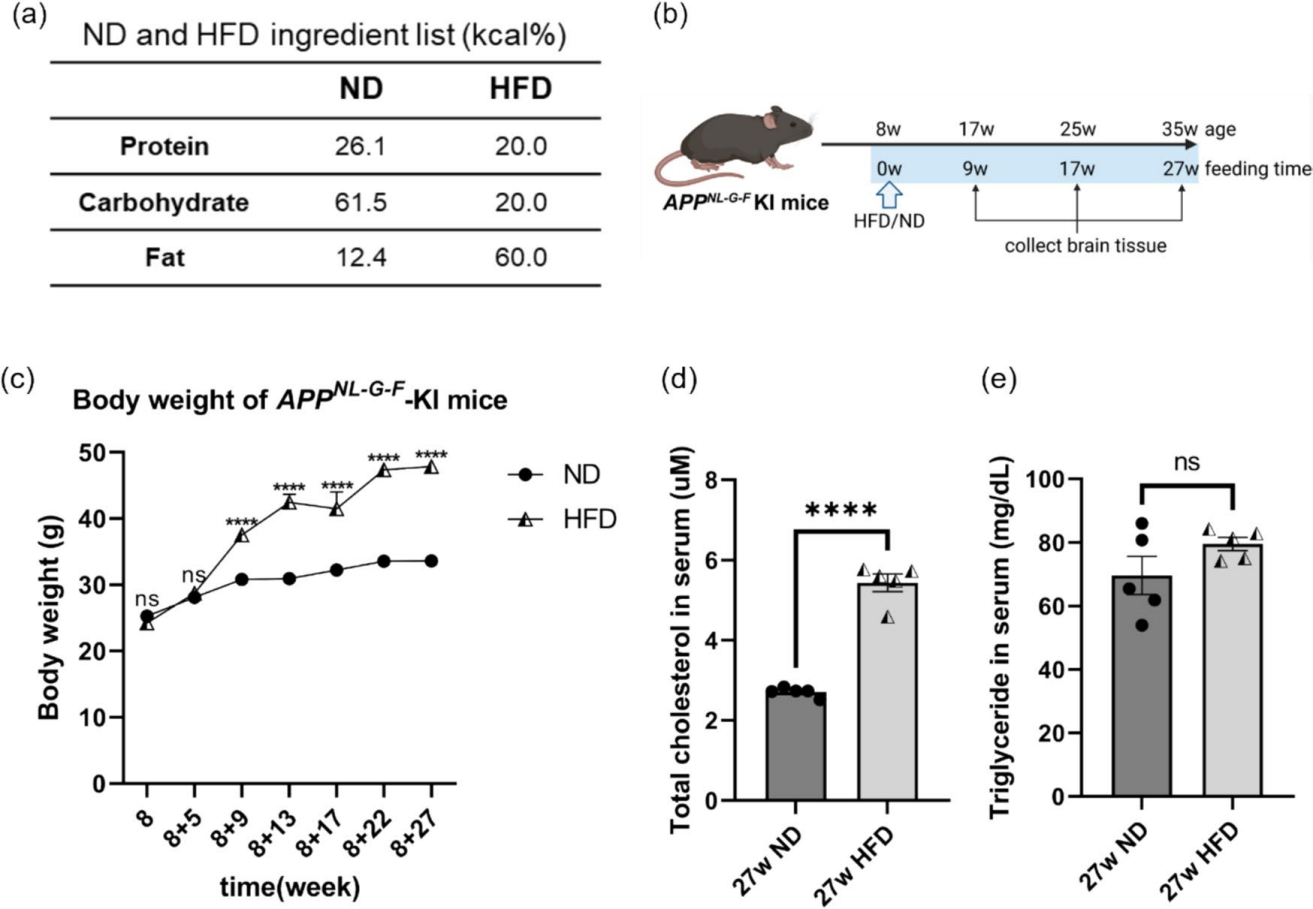
An obesity model was established in male *APP*^*NL–G–F*^ mice via HFD feeding. (**a**) Nutritional composition of the ND and the D12492 research diet (HFD) used in this study. (**b**) Schematic timeline showing the HFD or ND feeding in *APP*^*NL–G–F*^ mice. After weaning, all mice were maintained on ND until 8 weeks of age and then randomly assigned to either ND or HFD group. Mice in the ND group continued the same diet, while the HFD group was fed D12492-HFD. (**c**) Graph showing the body weight of *APP*^*NL–G–F*^ mice over the feeding time. Each dot represents the average weight of the *APP*^*NL–G–F*^ mice measured, *n* = 5 mice per group. After 9 weeks of separate feeding, the body weight of *APP*^*NL–G–F*^ mice fed an HFD was significantly higher than that of mice fed an ND. (d-e) Serum total cholesterol levels and TG levels in *APP*.^*NL–G–F*^ mice after 27 weeks of HFD or ND feeding. Each dot represents one mouse, *n* = 5 mice per group. Values are average ± SEM. Statistical significance was determined using multiple unpaired t-tests (****p < 0.0001; ns, not significant)

*APP*^*NL−G−F*^ knock-in AD model mice (*APP*^*NL−G−F*^ mice), carrying the Arctic, Swedish, and Beyreuther/Iberian mutations [24], and their wild-type (WT) littermates were used in this study. Mice were housed under specific pathogen-free conditions with a 12-h light/dark cycle, and food and water were provided ad libitum. At 8 weeks of age, the mice were randomly assigned to either ND or HFD groups. Mice in the HFD group were given ad libitum access to the D12492 research diet containing 60% fat, 20% protein, and 20% carbohydrate. Whereas mice in the ND group were fed the MF diet from ORIENTAL YEAST ad libitum, containing 12.4% fat, 26.1% protein, and 61.5% carbohydrates (Fig. 1a). Feeding lasted for 9, 17, or 27 weeks. At the end of the feeding period, mice were euthanized, and brain tissues were harvested for subsequent analyses (Fig. 1b). All animal experiments were approved by the Ethics Committee for Animal Experimentation of Tohoku University Graduate School of Medicine (approval number: 2020MdA-127) and carried out according to the Guidelines for Animal Experimentation of the Tohoku University Graduate School of Medicine and under the law and notification requirements of the Japanese government.

### Y-maze Test

The test was performed in a Y-shaped maze with three identical arms at 120-degree angles of each other. Male littermate *APP*^*NL−G−F*^ mice fed either an HFD or an ND for 27 weeks were employed in this test. After entering the maze from one arm, the mice were allowed to freely explore the three arms."alteration"defined as entry into a different arm than the one previously visited, which was considered a correct response, whereas returning to the previous arm was considered an error. Each mouse had 7 min to explore the maze, and we used the ANY-maze Video Tracking Software 6.1 (Stoelting) to record the total number and order of arm entries and calculate the percentage of alteration. The total number of arm entries within 7 min was used to assess the level of spontaneous activity, and the alteration rate was used to assess spatial memory.

### Serum Total Cholesterol and Triglyceride Measurements

#### Serum Sample Preparation

After 27 weeks of HFD or ND feeding, *APP*^*NL−G−F*^ mice underwent terminal blood collection. Following deep anesthesia, 1 mL of blood was collected from the left ventricle. The blood samples were left at room temperature for 30 min to allow clotting, then centrifuged at 2,000 × g for 15 min at 4 °C. The supernatant was carefully aspirated and stored at − 80 °C until analysis.

#### Cholesterol Assay

Total cholesterol was measured using the Amplex® Red Cholesterol Assay Kit (Invitrogen™, Cat. No. A12216) following the manufacturer's instructions. Specifically, mouse serum samples were diluted 1:40 with Reaction Buffer. Subsequently, 50 μL of the diluted serum was added to each well of a 96-well black-walled, clear-bottom microplate (Corning®, Cat. No. 3603). A standard curve was prepared by adding 50 μL of cholesterol standard solutions (0–20 μM) to designated wells. Then, 50 μL of the reaction mixture containing horseradish peroxidase, cholesterol oxidase, and cholesterol esterase was added to each well. The plate was incubated at 37 °C for 30 min, protected from light. Fluorescence intensity was measured using a FlexStation® 3 microplate reader (Molecular Devices) with excitation at 540 nm and emission at 590 nm. Total cholesterol concentrations in the serum samples were calculated based on the standard curve.

#### Triglyceride Assay

Triglyceride (TG) in serum was measured using the Triglyceride Colorimetric Assay Kit (Cayman Chemical, Cat. No. 10010303) according to the manufacturer's instructions. Serum samples were diluted 1:3 with Standard Diluent Assay Reagent. Subsequently, 10 μL of each diluted sample was added to a transparent 96-well microplate. A standard curve was prepared by adding 10 μL of triglyceride standard solutions (0–200 mg/dL) to designated wells. Then, 150 μL of Triglyceride Enzyme Mixture was added to each well. The plate was incubated at room temperature for 1 h, protected from light. Absorbance was measured using a FlexStation® 3 microplate reader (Molecular Devices) at 540 nm. TG concentrations in the serum samples were calculated based on the standard curve.

### Histopathology

#### Preparation of Brain Sections

After perfusion fixation in 4% fresh paraformaldehyde (PFA), whole brains were removed and stored in 4% PFA overnight. The following day, PFA was replaced with 10% sucrose in PBS for 12 h, followed by incubation in 20% sucrose in PBS overnight. On the third day, the brains were transferred to 30% sucrose in PBS for 24 h, then embedded in Optimal Cutting Temperature Compound (O.C.T. Compound) and stored at −80 ℃ until use. For frozen sectioning, 10 μm-thick sections were cut on a microtome cryostat (Leica CM1950) and kept at −80 ℃ until use. For the z-stack imaging experiment, 40 μm-thick floating sections were prepared and maintained in PBS until use. In the current study, brain sections from Bregma + 0.7 mm to Bregma −1.5 mm were used for cortical analysis, and brain slices from Bregma −1.5 mm to Bregma −2.5 mm were used for hippocampal analysis.

#### Immunofluorescence Staining

After drying, the sections were rinsed in PBS for 10 min, then permeabilized with 0.3% Triton X-100 for 30 min, and rinsed three times with PBS before staining. For immunofluorescence staining, sections were blocked with 2% skim milk in PBS at room temperature for 1 h, and then incubated overnight at 4 °C with the following primary antibodies: BAN50 (1:500, Aβ_1–16_, Wako #017–26871), LAMP1 (1:200, BD Pharmingen™ Purified Rat Anti-Mouse CD107a, #553,792), IBA1 (1:500, Anti IBA1, Rabbit, Wako, #019–19741), GFAP Monoclonal Antibody (2.2B10) (1:500, Invitrogen #13–0300), CD68 (1:500, Rat Anti-Mouse CD68, BIO-RAD, #164433), TREM2 (1:200, Human/Mouse TREM2 Antibody, R&D Systems, MAB17291-100), Anti-Lipoprotein lipase antibody [5D2] (Abcam, Cat. No. ab93898). After primary antibody incubation, sections were washed three times with PBS and incubated for 1 h at room temperature with the appropriate fluorochrome-conjugated secondary antibodies diluted in PBS: Goat Anti-Rabbit IgG Antibody (Alexa Fluor® 488), Goat Anti-Mouse IgG Antibody (Alexa Fluor® 568), Goat Anti-Rabbit IgG Antibody (Alexa Fluor® 488), Labeled Goat Anti-Rat IgG Antibody (Alexa Fluor® 568), Labeled Goat Anti-Rabbit IgG Antibody (Alexa Fluor® 568), Goat Anti-Mouse IgG Antibody (Alexa Fluor® 405). In some experiments, DAPI was concurrently used as a counterstain during secondary antibody staining. After the secondary antibody incubation, the sections were washed three times with PBS, followed by a single wash with distilled water (DW), and then mounted with Fluoromount™ (Diagnostic BioSystems, #K024).

#### Thioflavin-S Staining

After drying, the sections were rinsed with PBS for 10 min and incubated with 0.1% Thioflavin-S (Thermo Scientific, #213,150,250, diluted with 50% ethanol) for 5 min at room temperature. The sections were then rinsed with 70% ethanol for 5 min, washed twice with DW, and immersed in PBS for subsequent staining.

#### X34 Staining

After drying and permeabilization, sections were incubated with 10 μM X34 (diluted in 40% ethanol) at room temperature for 10 min and then differentiated with 0.2% NaOH (in 80% ethanol) for 2 min. After washing once with DW, sections were immersed in PBS for further staining.

#### BODIPY Staining of Floating Brain Sections

For LD staining, detergents were avoided, and thus Triton X-100 treatment was omitted. Floating 40 μm sections were incubated with the primary antibody (dilution 1:200) at 4 °C for 48 h. After the secondary antibody staining, the sections were stained with BODIPY™ 493/503 (1:1000) at room temperature for 15 min, ensuring the slices remained moist throughout.

#### Quantitative Analysis of Histopathology Data

Images were acquired using an Oxford Andor BC43, Keyence BZ-X800, or ZEISS LSM800 and analyzed with ImageJ or Zeiss Imaris software.

### Flow Cytometry

To investigate the expression level of TREM2 in microglia of the mouse brain, we used the Adult Brain Dissociation Kit (Miltenyi Biotec #130–107–677) to isolate single cells from the cortex and hippocampal tissues of *APP*^*NL−G−F*^ mice fed an ND or an HFD for 27 weeks. After removing red blood cells and debris, cells were blocked with Fc Receptor Blocking Reagent (1: 100) for 15 min at room temperature and then stained with PE anti-mouse/human CD11b Antibody (1:50, BioLengend, Cat. No. 101208, Clone M1/70), Alexa Fluor 488 anti-mouse CD45 Antibody (1:50, BioLengend, Cat. No. 103122, Clone 30-F11) and Human/Mouse TREM2 APC-conjugated Antibody (R&D Systems, Catalog #FAB17291 A) for 30 min at room temperature. After two washes with Phosphate Buffer, CD11b^+^ CD45^+^ microglia were sorted by flow cytometry to measure TREM2 expression levels.

Expression of TREM2 in OA-treated MG6 was also determined by flow cytometry. Briefly, OA-treated MG6 cells were incubated with CD16/CD32 monoclonal antibody (mAb) (2.4G2) for Fc receptor blocking and stained with Human/Mouse TREM2 APC-conjugated Antibody (R&D Systems, Catalog #FAB17291 A) for 20 min on ice followed by two washes with Phosphate Buffer. The fluorescence intensity of TREM2 was then measured by flow cytometry.

Data were acquired using a FACS CantoII analyzer (BD Biosciences) and analyzed with FLOWJO software (Tree Star, Ashland, Ore, USA).

### Lipid Composition Analysis of Cortical Tissue

After sacrificed, the whole brain was rapidly removed and the cortical tissue was dissected on ice, rinsed with cold PBS and promptly transferred to a −80 °C until use. Lipidomic analysis was performed by Human Metabolome Technologies, Inc. using liquid chromatography-mass spectrophotometry (LC–MS/MS). The detected lipid content is represented by the peak area, and the relative abundance of each lipid is represented by the relative peak area. For cluster analysis, the relative peak areas of lipids were standardized by z-score and then analyzed using SangerBox 3.0 (http://www.sangerbox.com/home.html) [25].

### Cholesterol Assay of Isolated Microglia

Single-cell suspensions were prepared from cortical tissues of *APP*^*NL−G−F*^ mice fed either an ND or an HFD for 27 weeks using the Adult Brain Dissociation Kit (Miltenyi Biotec, #130–107–677) as described in Sect."Flow Cytometry". After myelin removal, erythrocyte lysis and Fc receptors blocking, the cell suspension (about 2 × 10⁶ cells in 90 μL MACS buffer) was then incubated with 2 μL CD11b (Microglia) MicroBeads (Miltenyi Biotec, #130–093–636) for 15 min at 4 °C in the dark, then Magnetic-Activated Cell Sorting (MACS) was performed to isolate microglia. Next, aliquots of 5 × 10^4^ cells per sample were taken for cholesterol assay. Cells were lysed in the Reaction Buffer at room temperature for 5 min, centrifuged to remove debris, and the supernatants used for the assays. Total cholesterol was measured following the protocols outlined in Sect."Cholesterol Assay"; free cholesterol was measured by omitting cholesterol esterase, and ChE level calculated by subtracting free cholesterol from total cholesterol.

### Cell Culture and Treatment

A c-Myc-immortalized microglial cell line (MG6) derived from C57BL/6 WT mice was used for this in vitro study. Cells were maintained in Dulbecco’s modified Eagle’s medium (DMEM) containing 10% fetal bovine serum (FBS),1% L-glutamine, 1% penicillin–streptomycin, 10 μg/mL insulin, and 100 μM 2-mercaptoethanol (198–15,781, Wako, Osaka, Japan) in a CO_2_ incubator. Cells were treated with fatty acids (FAs) before staining or uptake experiments. Specifically, glass coverslips were placed in 24-well plates and coated with 500 μL of 50 g/mL poly-L-lysine at room temperature for 5 min and washed two times with DW. After drying, MG6 cells were split into wells and cultured overnight. The following day, cells were treated with oleic acid (OA), linoleic acid (LA), palmitic acid (PA), stearic acid (SA), or α-linolenic acid (ALA), each conjugated with 1% BSA, in culture medium supplemented with 5% FBS; control cells were maintained in medium containing 5% FBS and 1% BSA for the same duration. For Acyl-CoA:Cholesterol Acyltransferase inhibitor (ACAT) inhibitor treatment, MG6 microglial cells were pretreated with 50 μM Sandoz 58–035 (CAS No. 78934–83-5) for 12 h, followed by co-treatment with Sandoz 58–035 and OA for an additional 24 h.

### Cholesterol Assay of MG6 Microglia

MG6 microglia were treated with 100 μM OA for 24 h, either alone or together with 50 μM Sandoz 58–035, while untreated cells served as controls. Following trypsinization, cells were collected, counted, and aliquots of 5 × 10^4^ cells per sample were taken for cholesterol assay as described in Section"Cholesterol Assay of Isolated Microglia".

#### Fluorescence Staining of Cells

After treatment, MG6 cells were washed three times with D-PBS, fixed with 4% PFA on ice for 10 min, and washed three times with PBS. For LD staining, cells were incubated with BODIPY 493/503 (1:2000 from a 1 mg/mL stock solution in DMSO; Thermo Fisher) for 30 min at room temperature. For quantification, images were captured with an Oxford Andor BC43 using a 40 × Plan Fluorite dry objective and the total cell number and BODIPY^+^ LDs were analyzed using Zeiss Imaris software.

#### Aβ Peptides Uptake Assay

After treatment, MG6 cells were washed three times with D-PBS, and 0.5 nmol of Aβ peptides (Beta-Amyloid (1–42), HiLyte™ Fluor 555-labeled, #AS-60480–01) were added to each well. Following a 3-h incubation, cells were washed three times with D-PBS and subjected to subsequent fixation and staining. For quantification, images were captured with an Oxford Andor BC43 using a 40 × Plan Fluorite dry objective and analyzed using Zeiss Imaris software.

#### RT-qPCR

To purify total RNA from MG6 microglia, an RNeasy Micro Kit (Qiagen, #74,004) was used according to the manufacturer’s protocol. cDNA synthesis was performed with the GeneAce cDNA Synthesis Kit (NIPPON GENE #319–08881). RT-qPCR was performed using THUNDERBIRD SYBR qPCR Mix (TOYOBO, Osaka, Japan) on a 7500 Real-Time PCR System (Thermo Fisher Scientific). Relative gene expression was calculated by the ΔCt method and normalized to the amount of Gapdh. The primers we use are as follows: *Gapdh* (FASMAC, Forward, 5'-AGGTCGGTGTGAACGGATTTG-3'; Reverse, 5′-GGGGTCGTTGATGGCAACA-3′); *Trem2* (Eurofins, Forward, 5’-CTGGAACCGTCACCATCACTC-3’; Reverse, 5’-CGAAACTCGATGACTCCTCGG-3’).

#### RNA Sequencing and Data Analysis

RNA extracted from OA-treated and control MG6 cells with RNeasy Micro Kit (Qiagen, #74,004) was sent to the Bioengineering Laboratory for RNA sequencing. Cluster analysis and subsequent pathway enrichment analysis were performed on iDEP2.01 (https://bioinformatics.sdstate.edu/idep/) with raw count data from OA-treated and the control group. After TPM normalization, gene set enrichment analysis (GSEA) was performed using GSEA 4.3.3 software according to the instructions [26, 27]. The following gene sets were applied: Hallmark gene sets [28]; GOBP_CHOLESTEROL_EFFLUX (Systematic name: MM6818, Exact source: GO:0033344); GOBP_PHAGOCYTOSIS (Systematic name: MM4904, Exact source: GO:0006909); GOBP_PHAGOCYTOSIS_ENGULFMENT (Systematic name: MM16125, Exact source: GO: 0006911) [29].

#### Statistical Analyses and Reproducibility

All data represent the mean ± Standard Error of the Mean (S.E.M) from at least three independent experiments. Statistical comparisons of means were performed using GraphPad Prism 9. Unpaired student’s t-test was used for comparisons between two groups, and two-way ANOVA was employed to assess the main and interactive effects of diet and genotype in lipidomic analysis. Statistical significance was set at *p* < 0.05.

## Results

### HFD Intake Aggravated Aβ Deposition in *APP*^*NL−G−F*^ Mice Brain

8-week-old male *APP*^*NL−G−F*^ mice were fed either an ND or an HFD (Fig. 1a-b), and their body weights were recorded (Fig. 1c). Initially, both groups had similar weights; but after 9 weeks, the HFD group showed significantly higher body weights than the ND group. Thereafter, the HFD group consistently exhibited significantly higher body weights than the age-matched ND group (Fig. 1c). Additionally, serum total cholesterol and TG levels were measured in *APP*^*NL−G−F*^ mice after 27 weeks of HFD or ND feeding. Although HFD feeding did not significantly affect serum TG level (Fig. 1e), it markedly increased total cholesterol (Fig. 1d), demonstrating that prolonged HFD intake induced obesity and dyslipidemia in *APP*^*NL−G−F*^ mice.

**Fig. 2:**
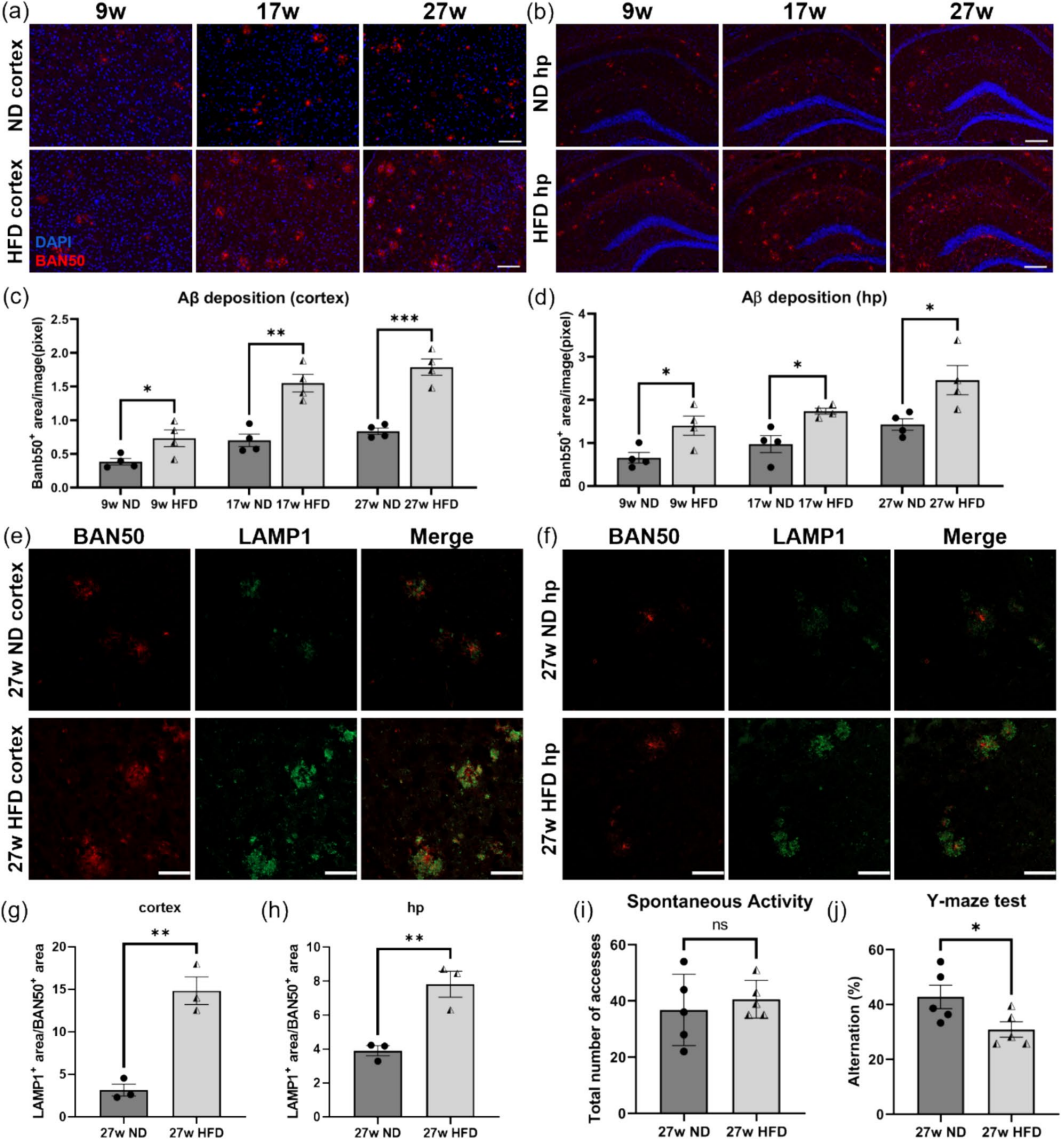
HFD feeding aggravated Aβ deposition, neurite dystrophy and memory impairment in *APP*^*NL–G–F*^ mice brain. (**a**-**b**) Representative images of brain sections from *APP*^*NL–G–F*^ mice fed an ND or an HFD for 9, 17, 27 weeks. Sections were stained with DAPI (blue) and BAN50 antibody against Aβ_1–16_ (red). In (**a**), images of the somatosensory cortex are shown (scale bar = 100 μm); in (**b**), images of the hippocampus are shown (scale bar = 200 μm). (**c**) Quantification of BAN50^+^ Aβ plaque area (in pixels) per image in the somatosensory cortex. 20 images were taken from each mouse brain for cortical analysis (*n* = 4 mice per group). (**d**) Quantification of BAN50^+^ Aβ plaque area (in pixels) per image in the hippocampus. 8 images were taken from each mouse brain for hippocampal analysis (*n* = 4 mice per group). (**e**–**f**) Representative images of brain sections from *APP*^*NL–G–F*^ mice fed with ND or HFD for 27 weeks, immunostained with LAMP1 antibody (green) and BAN50 (red). Scale bar = 50 μm. (**g**-**h**) Quantification of the LAMP1^+^ area/BAN50^+^ area ratio in the somatosensory cortex and hippocampus. 20 images were taken from each brain for cortical analysis and 12 images were taken from each brain for hippocampal analysis (*n* = 3 mice per group). (**i**) Y-maze tests were performed on *APP*.^*NL–G–F*^ mice fed an HFD for 27 weeks and their ND controls. The spontaneous activity level of mice was assessed as the total number of entries into all three arms within 7 min. (**j**) Mice spatial memory was evaluated by the alternation percentage, defined as the ratio of entries into the novel arm to the total number of arm entries (*n* = 5 mice per group). Values are average ± SEM. Statistical significance was determined using unpaired t-tests (*p < 0.05, **p < 0.01, ***p < 0.001; ns, not significant)

To explore the effects of HFD intake on Aβ-related pathological processes in *APP*^*NL−G−F*^ mouse brains, we selected the somatosensory cortex and hippocampus for a series of histopathological examinations as these areas are closely associated with motor, episodic, and spatial memory [30–32]. We performed immunofluorescence staining targeting Aβ_1–16_ in *APP*^*NL−G−F*^ mouse brain sections, and the results revealed that the amount of Aβ deposits in the somatosensory cortex and hippocampus increased with age in both dietary groups (Fig. 2a-b). Notably, Aβ deposition in mice fed with HFD for 9, 17 and 27 weeks was significantly higher than that of age-matched ND group (Fig. 2a-d), indicating that HFD feeding aggravated Aβ burden in *APP*^*NL−G−F*^ mice brains. The deposition of Aβ leads to local impairment of retrograde axonal transport and further maturation of lysosome precursors within swollen axons, resulting in their extensive accumulation around the plaques [33]. Thus, we labeled these swollen neurites with lysosome-associated membrane protein 1 (LAMP1) antibody, and the results showed that the area ratios of LAMP1^+^ to BAN50^+^ in the cortex and hippocampus of *APP*^*NL−G−F*^ mice fed an HFD for 27 weeks were significantly higher than those in the ND group, indicating that long-term HFD feeding resulted in more severe neurite dystrophy (Fig. 2e-h). To explore whether neuropathological deterioration led to impaired cognitive function, we next assessed the spatial working memory of *APP*^*NL−G−F*^ mice using the Y-maze test. The total number of entries into the arms within a specified time was used to measure spontaneous activity levels, and the results showed that HFD did not affect the spontaneous activity level of *APP*^*NL−G−F*^ mice (Fig. 2i) but significantly downregulated their spontaneous alternation percentage (Fig. 2j), indicating impaired spatial memory. In summary, HFD aggravated Aβ-related pathology and worsened cognitive impairment in *APP*^*NL−G−F*^ mice.

### HFD Intake Decreased Dense-core Aβ Plaque Formation By Reducing Microglial Recruitment to the Plaques

In AD brains, both astrocytes and microglia become activated in response to Aβ plaque formation and are recruited to the vicinity of the plaques to engulf Aβs [34]. To observe the positional relationship between astrocytes, microglia and plaques in our model, we conducted immunofluorescence staining to Aβ plaques as well as activated astrocytes and microglia. In the cortex of *APP*^*NL−G−F*^ mice fed either ND or HFD, almost all Aβ plaques were surrounded by IBA1^+^ microglia, whereas only a portion of plaques were surrounded by GFAP^+^ astrocytes, which were not as close to the plaques as microglia (Fig. S1a). Although astrocytes were denser in the hippocampus than in the cortex, they were still not in close contact with Aβ plaques (Fig. S1b). Based on the above observations, we hypothesized that microglia might play a major protective role in the model used in this study; therefore, we next focused on the effects of HFD on microglial function under AD pathological conditions.

**Fig. 3:**
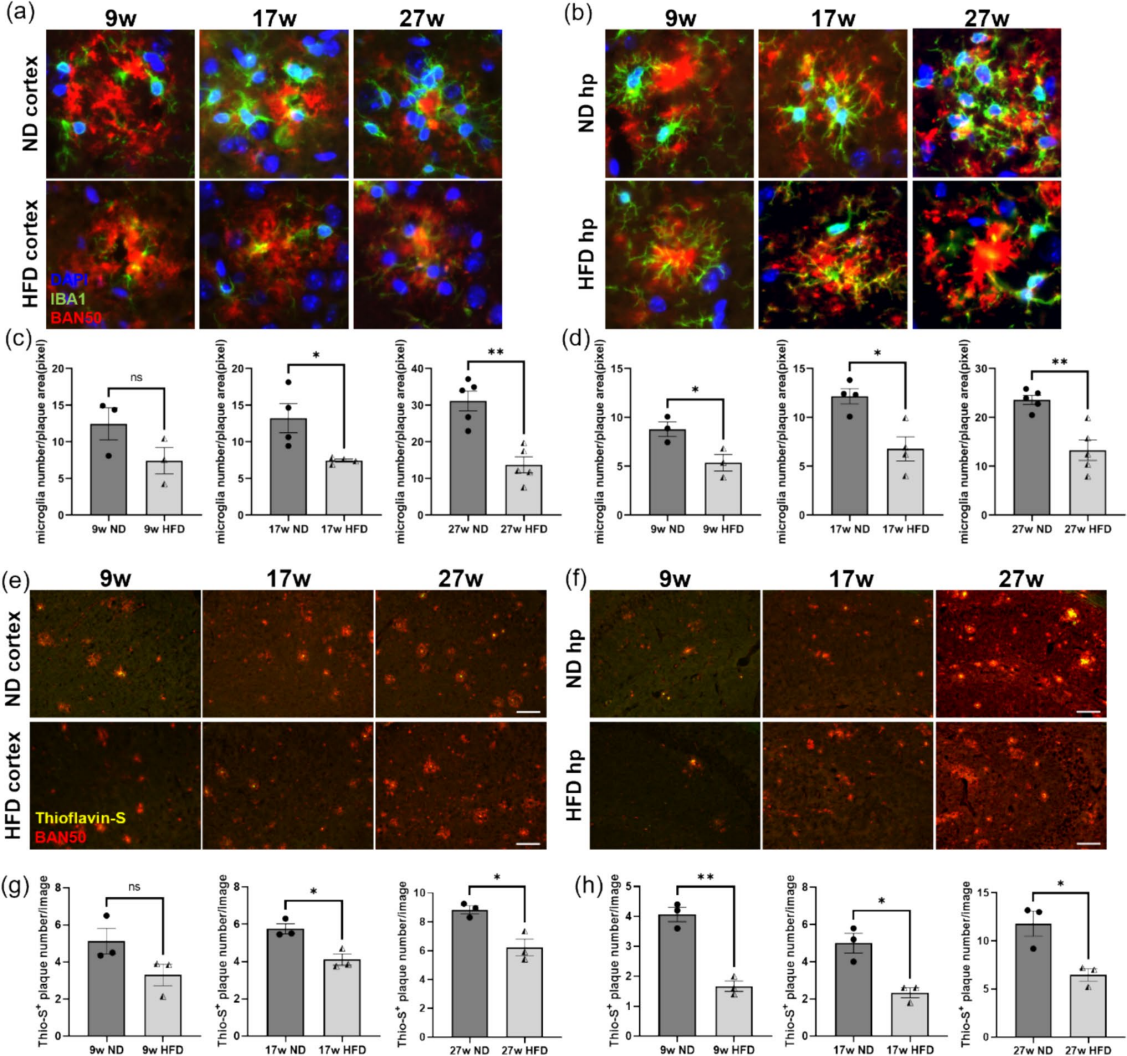
HFD feeding reduced the number of activated microglia around Aβ plaques and decreased dense-core plaque formation. (**a**-**b**) Representative images of the cortex and hippocampus from *APP*^*NL–G–F*^ mice fed with ND or HFD for 9, 17, 27 weeks. Sections were stained with BAN50 (red), IBA1 (green) and DAPI (blue) (image size: 80*80 μm^2^). (**c**) Quantification of IBA1^+^ microglia number around Aβ plaques in the cortex. Each dot represents one mouse, with 20 plaques analyzed per mouse (*n* = 3–5 mice per group). (**d**) Quantification of IBA1^+^ microglia number around Aβ plaques in the hippocampus. Each dot represents one mouse, with 10 plaques analyzed per mouse (*n* = 3–5 mice per group). (**e**–**f**) Representative images of cortex and hippocampus from *APP*^*NL–G–F*^ mice fed an ND or an HFD for 9, 17, 27 weeks, stained with Thioflavin-S (yellow) and BAN50 (red). Scale bar = 100 μm. (**g**) Quantification of the average number of Thioflavin-S^+^ dense-core plaques per image in the cortex. Each dot represents one mouse, with 20 images analyzed per mouse (*n* = 3 mice per group). (**h**) Quantification of the average number of Thioflavin-S.^+^ dense-core plaques per image in the hippocampus. Each dot represents one mouse, with 5 images analyzed per mouse (*n* = 3 mice per group). Values are average ± SEM. Statistical significance was determined using an unpaired t‐test (*p < 0.05, **p < 0.01; ns, not significant)

Microglial morphological dystrophies were specifically detected in the hypothalamus of postmortem brains from obese individuals [35]. Single-cell RNA sequencing also revealed that HFD dysregulated cell-to-cell communication in hippocampal microglia of mice and altered the expression of genes related to endoplasmic reticulum stress and protein folding [36]. To investigate whether HFD affected microglial activation and recruitment to Aβ plaques in the brain of *APP*^*NL−G−F*^ mice, we quantified the number of microglia surrounding and interacting with individual plaques in the cortex and hippocampus of *APP*^*NL−G−F*^ mice. The results demonstrated that a 9-week HFD feeding resulted in a downward trend in microglial number around Aβ plaques in the cortex, though the reduction did not reach statistical significance. However, both 17-week and 27-week HFD feedings led to a marked decrease in microglial numbers around the plaques (Fig. 3a, c). Similarly, in the hippocampus, HFD significantly diminished microglial aggregation around the plaques (Fig. 3b, d). Microglia envelop Aβ plaques, acting as a physical barrier to restrict amyloid fibrils polymerization and to squeeze diffuse plaques into less toxic dense-core plaques; without this compacting effect, plaques would continue to spread and grow acceleration the progression of AD [17]. To confirm the association between microglia and dense-core plaque formation in *APP*^*NL−G−F*^ mice brain, we performed staining of microglia, Aβ, and dense-core Aβ plaques in the cortex of 35-week-old ND-fed *APP*^*NL−G−F*^ mice. As shown in Fig. S2, even within the same image, the morphology of Aβ plaques exhibits heterogeneity. Thioflavin-S^+^ BAN50^+^ densely compacted Aβ plaques were tightly surrounded by multiple IBA1^+^-activated microglia (Fig. S2b, yellow box), whereas there were fewer IBA1^+^ microglia around Thioflavin-S^−^ BAN50^+^ diffuse plaques (Fig. S2c, green box), indicating the indispensable role of microglia in the formation of dense-core plaques. To verify whether the reduction in microglial clustering Aβ plaques induced by HFD feeding led to a decreased formation of dense-core plaque formation, we quantified dense-core plaques in the cortex and hippocampus of *APP*^*NL−G−F*^ mice. Consistent with our hypothesis, dense-core plaques were significantly reduced in HFD-fed groups compared to age matched ND-fed groups, except for a nonsignificant decreasing trend in the cortex after 9 weeks of HFD feeding. (Fig. 3e-h). In essence, HFD feeding decreased the recruitment of microglia to Aβ plaques and attenuated the compaction of diffuse plaques into dense-core plaques, which may be one of the reasons for the widespread diffusion of Aβ plaque in the brains of the HFD-fed *APP*^*NL−G−F*^ mice.

### HFD Intake Reduced CD68 Expression in Microglia of *APP*^*NL−G−F*^ Mice Brain

**Fig. 4:**
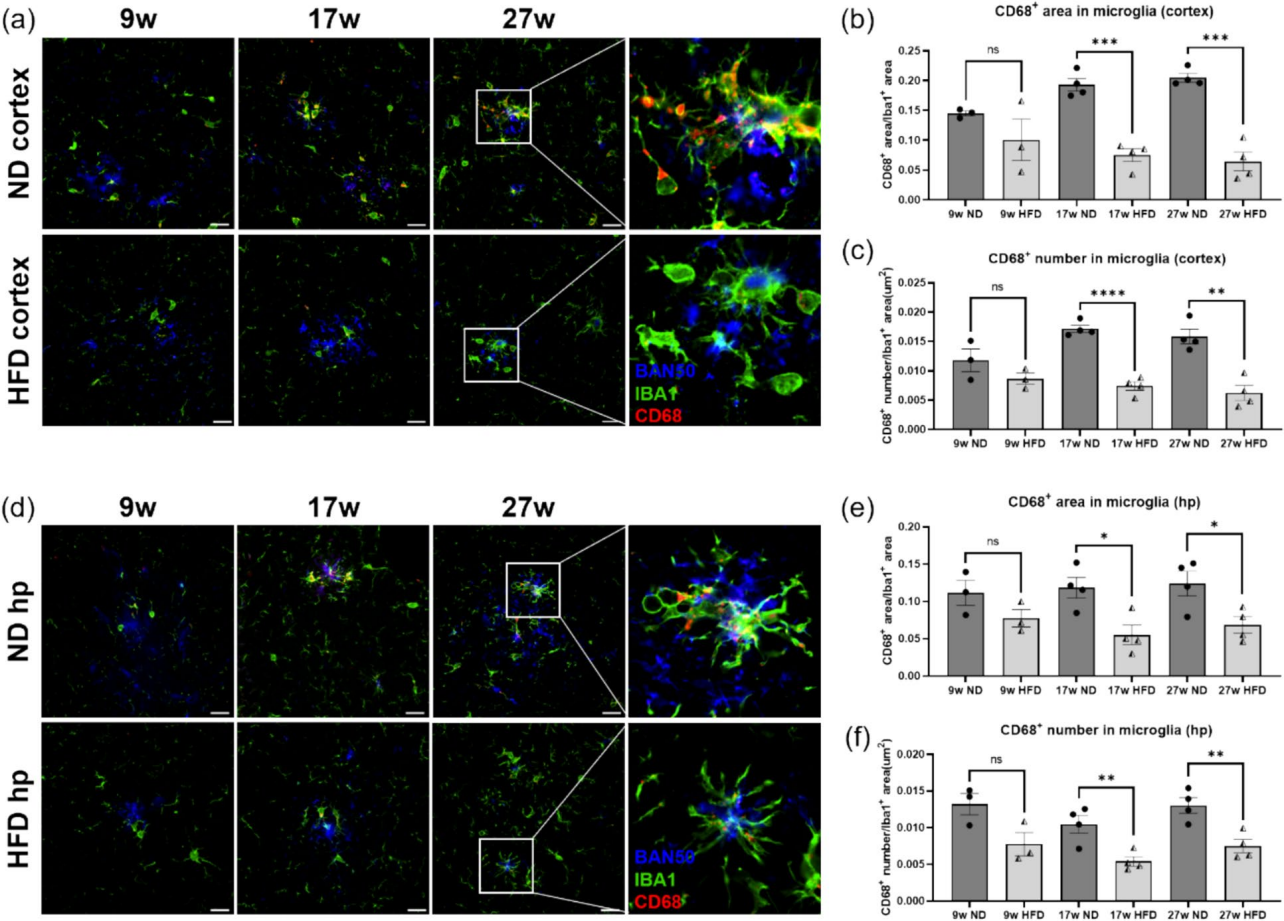
HFD feeding reduced CD68 expression in microglia of *APP*^*NL–G–F*^ mice brain. (**a**) Representative images of somatosensory cortex from *APP*^*NL–G–F*^ mice fed with ND or HFD for 9, 17, 27 weeks. Sections were stained with BAN50 (blue), IBA1 (green) and CD68 (red). Scale bar = 20 μm. The right panel shows a higher magnification image of the plaques and microglia in the white box from the 27-week fed group. (**b**-**c**) Quantification of area and number of discrete CD68^+^ puncta within IBA1^+^ microglia in somatosensory cortex. Each dot represents one mouse, with 20 images analyzed per mouse (*n* = 3–4 mice per group). (**d**) Representative images of hippocampus from *APP*^*NL–G–F*^ mice fed with ND or HFD for 9, 17, and 27 weeks. Sections were stained with BAN50 (blue), IBA1 (green) and CD68 (red). Scale bar = 20 μm. The right panel shows a higher magnification image of the plaques and microglia in the white box from the 27-week fed group. (**e**–**f**) Quantification of area and number of discrete CD68^+^ puncta within IBA1^+^ microglia in hippocampus. Each dot represents one mouse, with 10 images analyzed per mouse (*n* = 3–4 mice per group). Values are average ± SEM. Statistical analyses were performed using an unpaired t-test. Statistical significance was determined using an unpaired t-test (*p < 0.05, **p < 0.01, ***p < 0.001, ****p < 0.0001; ns, not significant)

The clearance of Aβ relies on the internalization by microglia. CD68 is a microglia/macrophage marker associated with lysosomal glycoproteins that shuttle in vesicles between lysosomes, endosomes, and the plasma membrane, and serves as a general marker for activated phagocytic microglia/macrophages [37, 38]. To assess whether HFD affected the phagocytic activity of microglia, we performed CD68 staining on microglia surrounding the plaques. We observed that CD68 was highly expressed in the cytoplasm of activated microglia near the Aβ plaques in the cortex and hippocampus of ND-fed *APP*^*NL−G−F*^ mice (Fig. 4a, d). Although 9 weeks of HFD feeding did not significantly alter CD68 expression in microglia, CD68 levels in these microglia were significantly lower in the cortex and hippocampus of mice fed HFD for 17 and 27 weeks than those in the ND group (Fig. 4b-c, 4e-f), suggesting that HFD could impair the ability of microglia to internalize and degrade Aβ.

### HFD Intake Led to an Increase in Cholesterol Esters in the Cerebral Cortex of WT and *APP*^*NL−G−F*^ Mice

**Fig. 5:**
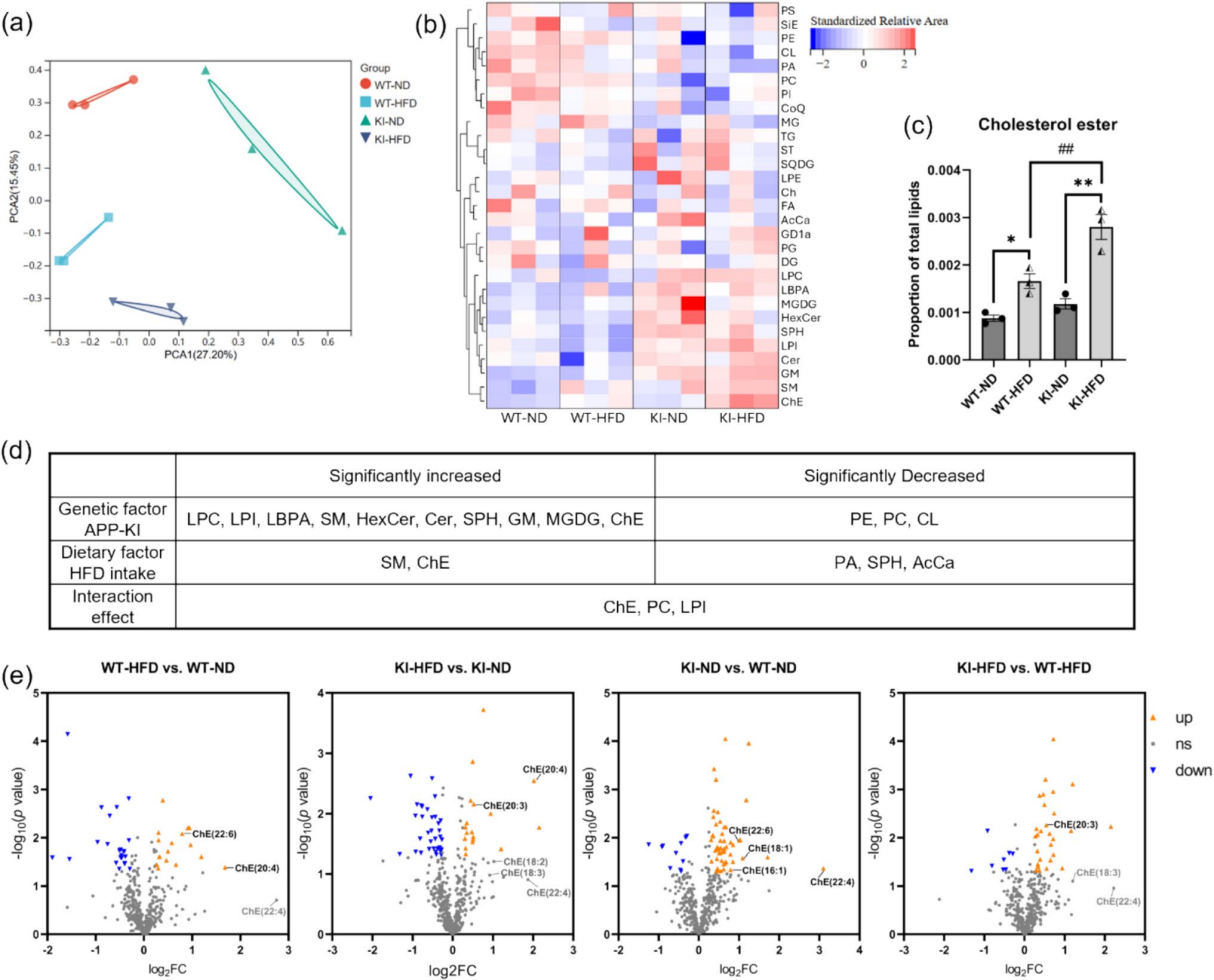
HFD feeding induced alterations in lipid composition and enrichment of ChE in mouse cerebral cortex tissue. (**a**) PCA score plot of lipids in cortical tissue. (**b**) Spearman correlation heatmap showing cluster analysis of major lipid classes. The peak area of each lipid class was standardized and represented by the blue–white–red (BWR) gradient color. (**c**) The proportion of ChE in the cortex of ND- or HFD-fed WT mice and *APP*^*NL−G−F*^ mice. Values are average ± SEM. Statistical analyses were performed using an unpaired t-test. *p < 0.05, **p < 0.01 (HFD vs. ND), ^##^*p* < 0.01 (*APP*^*NL−G−F*^ vs. WT). (**d**) A two-way ANOVA analysis summarized the effects of genotype and diet on lipid proportion. (**e**) Volcano plot illustrating fold-change and FDR-corrected *p*-values of all detected lipid species. Lipid species with a > 1.2-fold increase (log_2_ FC > 0.263) and *p* value < 0.05 (-log_10_ *p* > 1.3) are shown in orange triangles. Lipid species a > 1.2-fold decrease (log_2_ FC < −0.263) and *p* value < 0.05 (-log_10_ *p* > 1.3) are shown in blue triangles. Gray dots represent lipids with FC < 1.2 or no significant difference. Abbreviation: FC: Fold change. *n* = 3 mice per group

Microglia demonstrate metabolic plasticity, enabling them to rapidly adapt to changes in environmental energy availability and utilize alternative energy sources, however, their immune surveillance, phagocytic activity, and response to external stimuli may be altered [39, 40]. To investigate whether HFD induced microglial dysfunction by altering the lipid composition in the brain, we performed lipidomic analysis on cortical tissues from littermate *APP*^*NL−G−F*^ and WT mice fed an HFD or an ND. A total of 455 lipids were detected using LC–MS, and their relative proportions within the total lipid content were quantified. The Principal Component Analysis (PCA) plot showed distinct lipid composition patterns observed among the four groups (Fig. 5a). To explore how *APP*^*NL−G−F*^ gene knock-in and HFD feeding disturb the changes in lipids in cortical tissue, we clustered the major categories of these lipids (Fig. 5b, d). In the cerebral cortex, lysophosphatidylcholine (LPC), Lysophosphatidylinositol (LPI), lysobisphosphatidic acid (LBPA), hexosylceramide (HexCer), ceramide (Cer), sphingosine (SPH), monosialogangliosides (GM), sphingomyelin (SM), monogalactosyldiacylglycerol (MGDG) and cholesterol ester (ChE) were significantly upregulated due to the knock-in of *APP*^*NL−G−F*^ gene (Genetic factor shown in Fig. 5d). In comparison, only SM and ChE were significantly upregulated following long-term HFD intake (Dietary factor shown in Fig. 5d). Although SM constitutes a larger proportion in cortical tissue, the effects of dietary and genetic factors only caused a slight alteration in its relative abundance, with no significant differences observed among the four groups (Fig. S3b). In contrast, ChE proportion in the cerebral cortex dramatically increased following HFD intake in both WT and *APP*^*NL−G−F*^ mice (Fig. 5c). In addition to the individual effects of genotype and HFD, we observed a significant interaction between these two factors concerning the ChE proportion (Interaction effect shown in Fig. 5d), suggesting a unique characteristic of this lipid in the brains of obese *APP*^*NL−G−F*^ mice compared to other lipid species. Based on a *p*-value less than 0.05 and a fold change greater than 1.2, the volcano plot showed significant perturbation induced by the *APP*^*NL−G−F*^ gene knock-in and HFD feeding (Fig. 5e), indicating that the lipids upregulated by these two factors include several ChEs. Collectively, we report for the first time that long-term HFD intake upregulated multiple ChEs in the cerebral cortex of *APP*^*NL−G−F*^ mice, pointing to a synergistic effect of HFD on the accumulation of ChE in AD brain.

### HFD Induced Massive Aggregation of LDs in Microglia in the Cerebral Cortex of *APP*^*NL−G−F*^ Mice

**Fig. 6:**
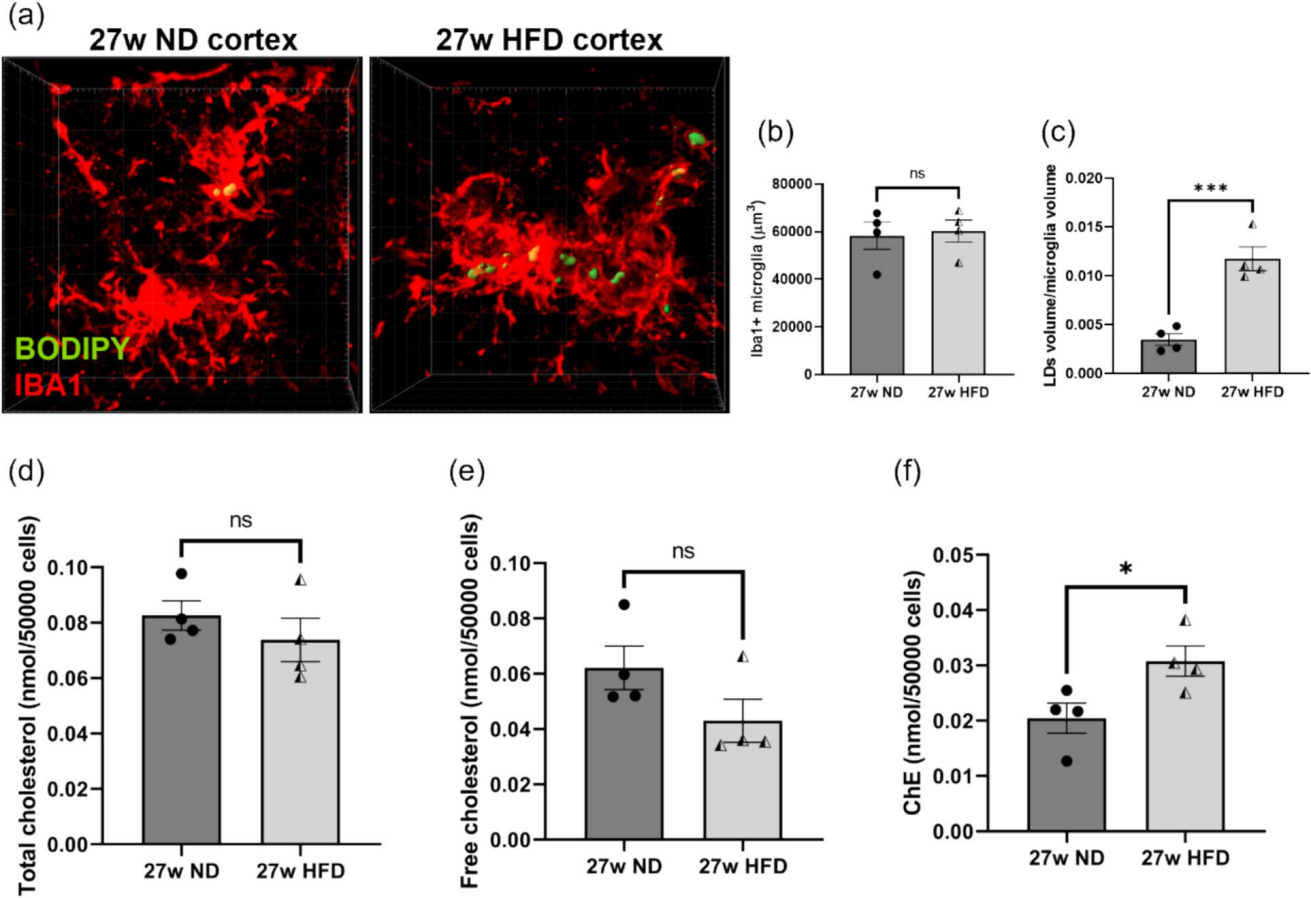
HFD feeding increased LDs accumulation in microglia of *APP*^*NL–G–F*^ mice brain. (**a**) Representative 3D images of BODIPY^+^ LDs in cortical microglia of *APP*^*NL–G–F*^ mice fed ND or HFD for 27 weeks. (**b**) The average volume of microglia in the images of mice cortex in the ND and HFD groups used for analysis. There was no significant difference in the volume of microglia analyzed between the two groups. (**c**) The volume of LDs accumulated per unit volume of microglia. After 27 weeks of HFD feeding, the accumulation of LDs in microglia was significantly higher than that in the ND control group. Each dot represents an analyzed mouse. 12 images were analyzed in each mouse cortex. *n* = 4 mice per group. (**d**-**f**) Total cholesterol, free cholesterol and ChE levels in isolated cortical microglia of *APP*.^*NL–G–F*^ mice fed ND or HFD for 27 weeks. Each dot represents an analyzed mouse, *n* = 4 mice per group. Values are average ± SEM. Statistical analyses were performed using an unpaired t-test (*p < 0.05, ***p < 0.001; ns, not significant)

ChE are formed by the esterification of excess cholesterol by ACAT and subsequently stored in intracellular LDs [41], therefore, we hypothesized that HFD feeding might also lead to the accumulation of LDs in microglia of *APP*^*NL−G−F*^ mice. We stained floating *APP*^*NL−G−F*^ brain sections with BODIPY and found massive LDs in activated microglia surrounding Aβ plaques (Fig. 6a) and then quantified the volume of activated microglia and the LDs within them. Although there was no significant difference in the average volume of IBA1^+^ microglia between the ND and HFD groups (Fig. 6b), the volume of LDs per unit volume of microglia in the cortex of HFD-fed mice was significantly higher than that in the ND group (Fig. 6c). Furthermore, microglia were isolated from the cortical tissue of *APP*^*NL−G−F*^ mice fed either an HFD or an ND for 27 weeks and subjected to cholesterol assay. Although total cholesterol and free cholesterol levels in microglia from HFD-fed mice was comparable to those in ND controls (Fig. 6d-e), ChE levels were markedly increased (Fig. 6f). These results indicate that HFD intake leads to the accumulation of ChE within microglia, stored as LDs. Formation and dynamic changes of LDs are intimately related to the activation state of microglia, and their excessive accumulation may precipitate disruptions in intracellular lipid metabolism, thereby triggering pro-inflammatory signaling pathways and exacerbating inflammatory responses [42, 43]. Therefore, we postulate that the impairment of microglial function in the brains of *APP*^*NL−G−F*^ mice induced by long-term HFD feeding may be attributed to the excessive accumulation of ChE and the overload of LD in microglia.

### In Vitro OA Treatment Induced LD Accumulation in MG6 and Downregulated their Phagocytic Activity

**Fig. 7:**
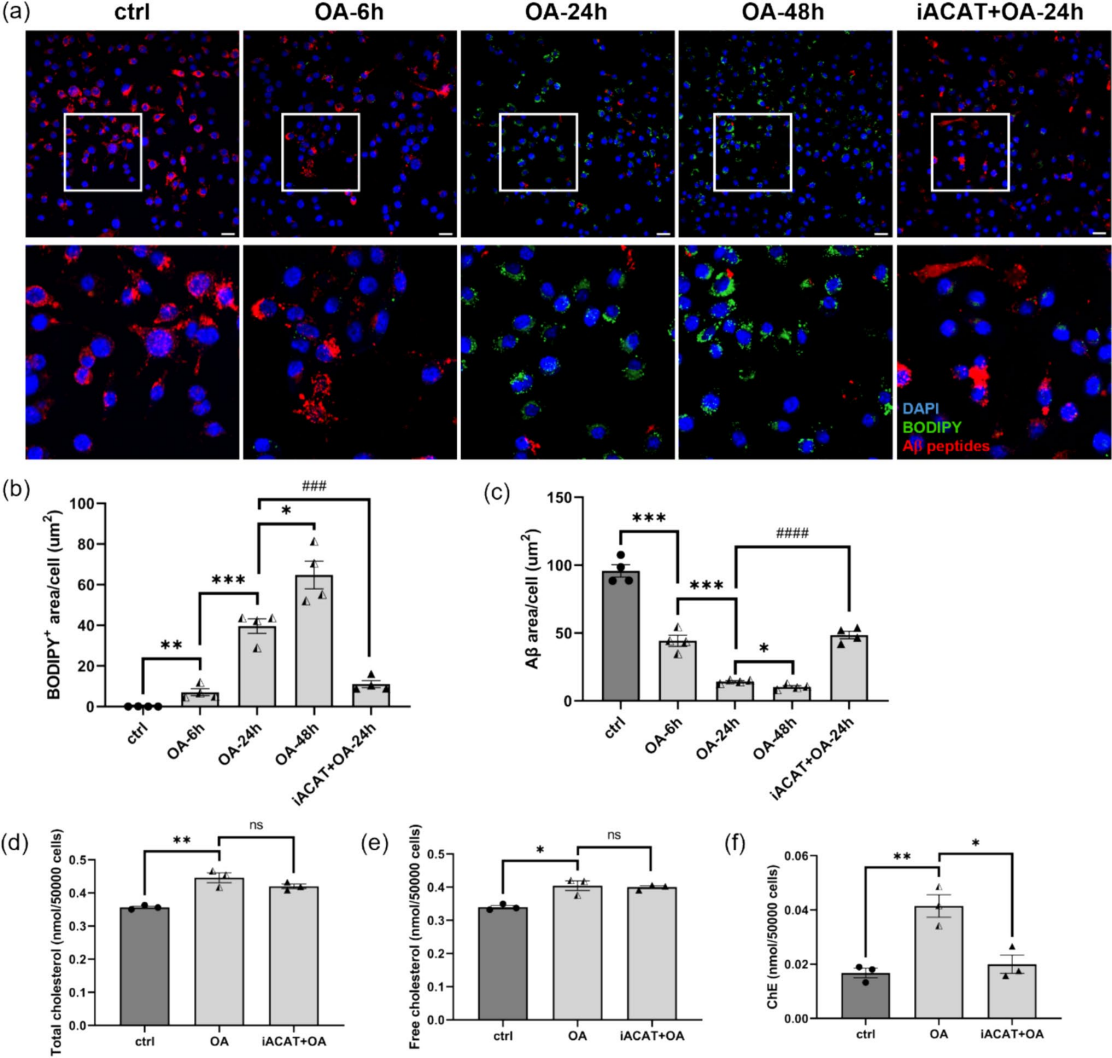
OA treatment induced LDs accumulation in MG6 microglia and reduced their phagocytic function. (**a**) Representative images of MG6 cells treated with 100 μM OA for 6, 24, or 48 h, or co-treated with 100 μM OA and 50 μM ACAT1 inhibitor Sandoz 58–035 (iACAT) for 24 h. Following treatment, cells were incubated with 1 μM of HiLyte™ Fluor 555-labeled Aβ_1–42_ for 3 h, then stained with BODIPY for LDs and DAPI for nuclei. Scale bars = 20 μm. The lower panel shows higher magnification views of MG6 microglia in the white boxed regions. (**b**-**c**) Quantification of area of BODIPY^+^ LDs per cell and Aβ peptides uptake by each MG6 microglia.. Each dot represents an independent experiment. Four independent experiments were performed for each group, with 8 images analyzed per experiment. (**d**-**f**) Total cholesterol, free cholesterol and ChE levels in MG6 microglial lysates following 24 h treatment with 100 μM OA or co-treatment with 100 μM OA and Sandoz 58–035. Each dot represents an individual experiment, and 3 individual experiments has been performed. Values are average ± SEM. Statistical analyses were performed using an unpaired t-test (*p < 0.05, **p < 0.01, ***, ^###^*p* < 0.001, ^####^*p* < 0.0001, ns, not significant)

Both the overall quantity and the specific composition of dietary FAs have been shown to exert direct modulatory effects on the physiological state of microglia [43]. The D12492-HFD used in this study comprises a substantial proportion of FAs, constituting 32.89% of its total weight. Within the overall FA composition, OA accounts for 34%, LA 29%, PA 20%, SA 10%, and ALA constitutes 2%. To investigate the individual effects of these FAs on microglial phenotypes, we treated MG6 microglial cells in vitro with each of the five FAs separately for 24 h, followed by Aβ uptake assay and BODIPY staining. Among these FAs, OA—the most abundant FA in the D12492-HFD—significantly promoted LD formation in MG6 microglia and reduced the uptake of HiLyte™ Fluor 555-labeled Aβ peptides (Fig. S4a-c). In contrast, the other four FAs did not promote LD accumulation nor significantly impair Aβ uptake in MG6 microglia (Fig. S4a-c). To further validate the effects of OA, MG6 microglia were treated with 100 μM OA for 6, 24, or 48 h. We observed that OA promoted intracellular LD accumulation and inhibited the uptake of Aβ in a time-dependent manner (Fig. 7a-c). To investigate whether OA-induced LD accumulation reflects an increase in ChE, MG6 microglia were treated with 100 μM OA with or without 50 μM Sandoz 58–035, a competitive inhibitor of ACAT1 (iACAT). Following OA treatment, total cholesterol levels increased, with significant elevations observed in both free cholesterol and ChE fractions, whereas co-treatment with Sandoz 58–035 only reduced ChE level (Fig. 7d-f), indicating that Sandoz 58–035 specifically inhibited the cholesterol esterification process. Importantly, co-treatment with Sandoz 58–035 and OA diminished LD formation while restoring Aβ peptides phagocytic activity in MG6 microglia (Fig. 7a-c), supporting the notion that OA-induced phenotypic alterations in MG6 were mediated through ChE accumulation.

### OA Treatment Downregulated Pathways Related to Microglial Cholesterol Homeostasis and Phagocytic Activity

**Fig. 8:**
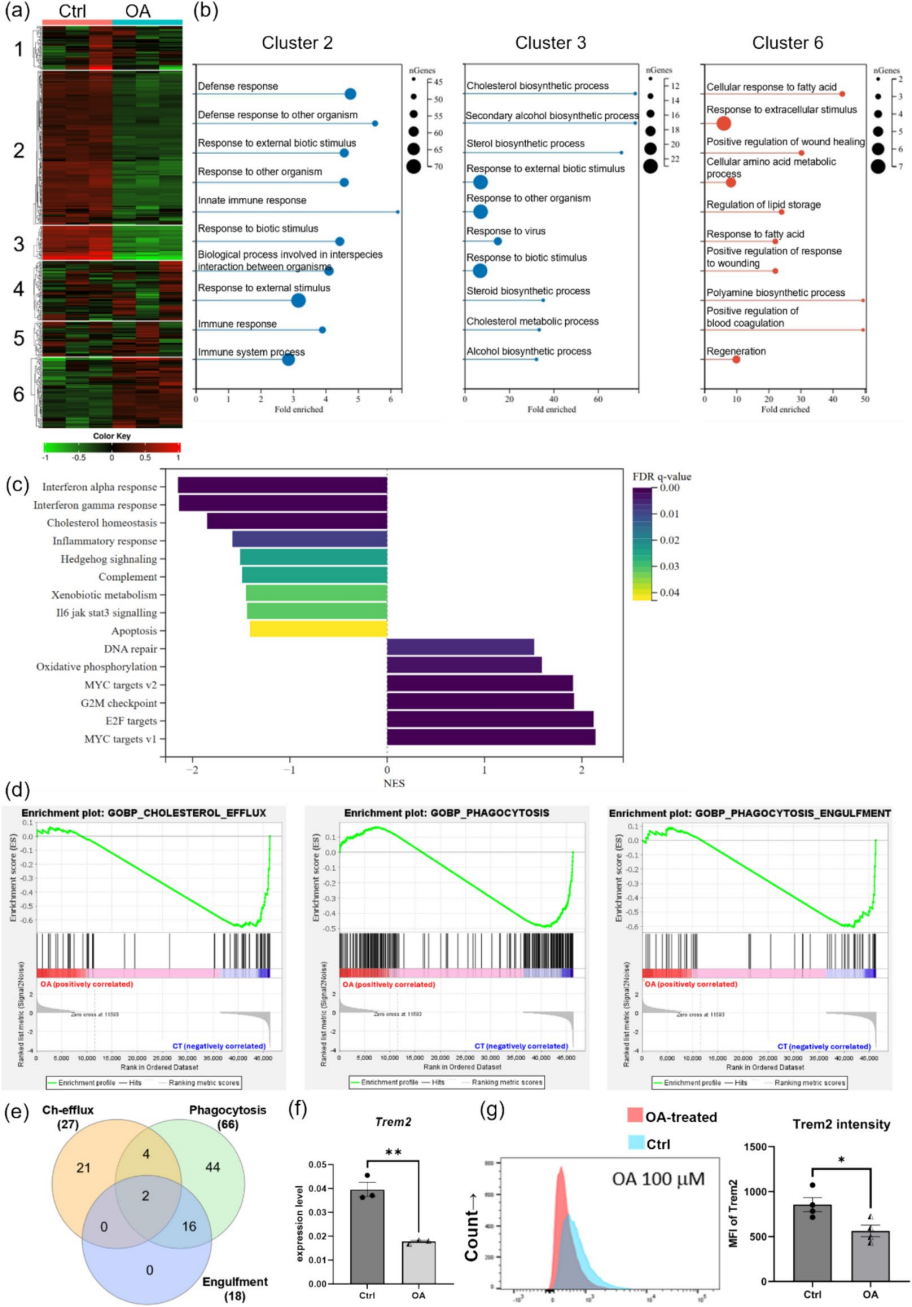
OA treatment altered the gene expression pattern of MG6 microglia. (**a**) Heatmap of k-means clustering of top 500 differentially expressed genes between OA-treated MG6 and control MG6. (**b**) GOBP analysis showing the major enriched pathways for genes in cluster2, 3, and 6 in the heatmap. (**c**) GSEA results showing gene sets significantly downregulated or upregulated in MG6 after OA treatment. Gene set database: MH collection: Hallmark gene sets. (**d**) GSEA results showing the enrichment of gene sets of interest in OA-treated versus control MG6 cells. Gene set 1: GOBP_CHOLESTEROL_EFFLUX (Normalized Enrichment Score [NES] = −1.4495167, FDR *q*-value = 0.013565891). Gene set 2: GOBP_PHAGOCYTOSIS (NES = −1.2518736, FDR *q*-value = 0.031307552). Gene set 3: GOBP_PHAGOCYTOSIS_ENGULFMENT (NES = −1.3222779, FDR *q*-value = 0.04536862). (**e**) Venn diagram of the leading-edge genes common to the three pathways in (**d**). (**f**) RT-qPCR results of *Trem2* gene expression level in MG6 cells treated with 100 μM OA for 24 h. Each dot represents an independent experiment (*n* = 3). (**g**) Representative flow cytometry histogram and quantification of TREM2 fluorescence in MG6 cells treated with 100 μM OA for 24 h. Each dot represents an independent experiment (*n* = 4). Values are average ± SEM. Statistical significance was determined using an unpaired t-test (*p < 0.05, **p < 0.01)

In vitro, OA-treated MG6 cells exhibit a phenotype similar to that of microglia in the brains of *APP*^*NL−G−F*^ mice fed an HFD, characterized by extensive intracellular accumulation of LDs and reduced phagocytic activity. To investigate how OA treatment perturbed microglial gene expression, we performed RNA sequencing analysis on MG6 treated with OA for 24 h compared with control MG6 cells. As shown in Fig. 8a, the heatmap highlights the top 500 differentially expressed genes (DEGs), which were partitioned into six clusters via k-means clustering analysis. Among the six clusters, genes in clusters 2 and 3 were downregulated by OA treatment, whereas genes in cluster 6 were upregulated. We separately listed the top 10 statistically significant Gene Ontology (GO) biological process (BP) terms from clusters 2, 3, and 6 (Fig. 8b). Among them, OA downregulated several processes related to immune response, defense response, as well as cholesterol, sterol biosynthesis, and cholesterol metabolism. While it upregulated processes associated with cellular response to FA and lipid storage. To further confirm which pathways in OA-treated MG6 cells were significantly regulated, we performed Gene Set Enrichment Analysis (GSEA) using the RNA sequencing results. Consistent with the clustering analysis, analysis using the mouse-ortholog hallmark gene set database revealed that nine gene sets were significantly downregulated by OA treatment, including those associated with interferon response, cholesterol homeostasis, and inflammatory response (Fig. 8c). In addition, we selected gene sets related to cholesterol metabolism and phagocytosis for enrichment analysis and found that OA significantly downregulated the processes of cholesterol efflux, phagocytosis, and engulfment (Fig. 8d). The preceding results align with the phenotype observed in OA-treated MG6 cells. The excessive accumulation of LDs is likely associated with the downregulation of genes involved in cholesterol efflux and the upregulation of lipid storage genes, which may lead to reduced microglial reactivity and phagocytic activity.

The leading-edge genes in the three gene sets (cholesterol efflux, phagocytosis, and engulfment) downregulated by OA treatment encompass several AD risk genes (Fig. 8d). Of note, six genes, including triggering receptor expressed on myeloid cells 2 (*Trem2*), are concurrently present in the leading-edge genes of both cholesterol efflux and phagocytosis gene sets (Fig. 8e; genes highlighted in red in Fig. S5). *Trem2* not only functions as one of the receptors for Aβ in microglia but also regulates their activation state and functional phenotype in both physiological conditions and various disease contexts, critically driving the transition to the fully activated disease-associated microglia (DAM) phenotype in the AD brain. [18, 19, 45, 46]. Mutations in *Trem2* lead to an impaired response to initial stimuli and disruptions in lipid metabolism [23, 47]. To further investigate the effect of OA treatment on the expression of *Trem2* in microglia, we then performed RT-qPCR test on MG6 in OA-treated and control groups, and the results showed a significant downregulation of *Trem2* levels following OA treatment (Fig. 8f). In addition, the analysis results of MG6 labeled with TREM2 antibody by flow cytometry also showed that OA downregulated the expression of TREM2 on MG6 cells (Fig. 8g).

### HFD Intake Downregulated TREM2 Expression of Microglia in *APP*^*NL−G−F*^ Mice Brain

**Fig. 9:**
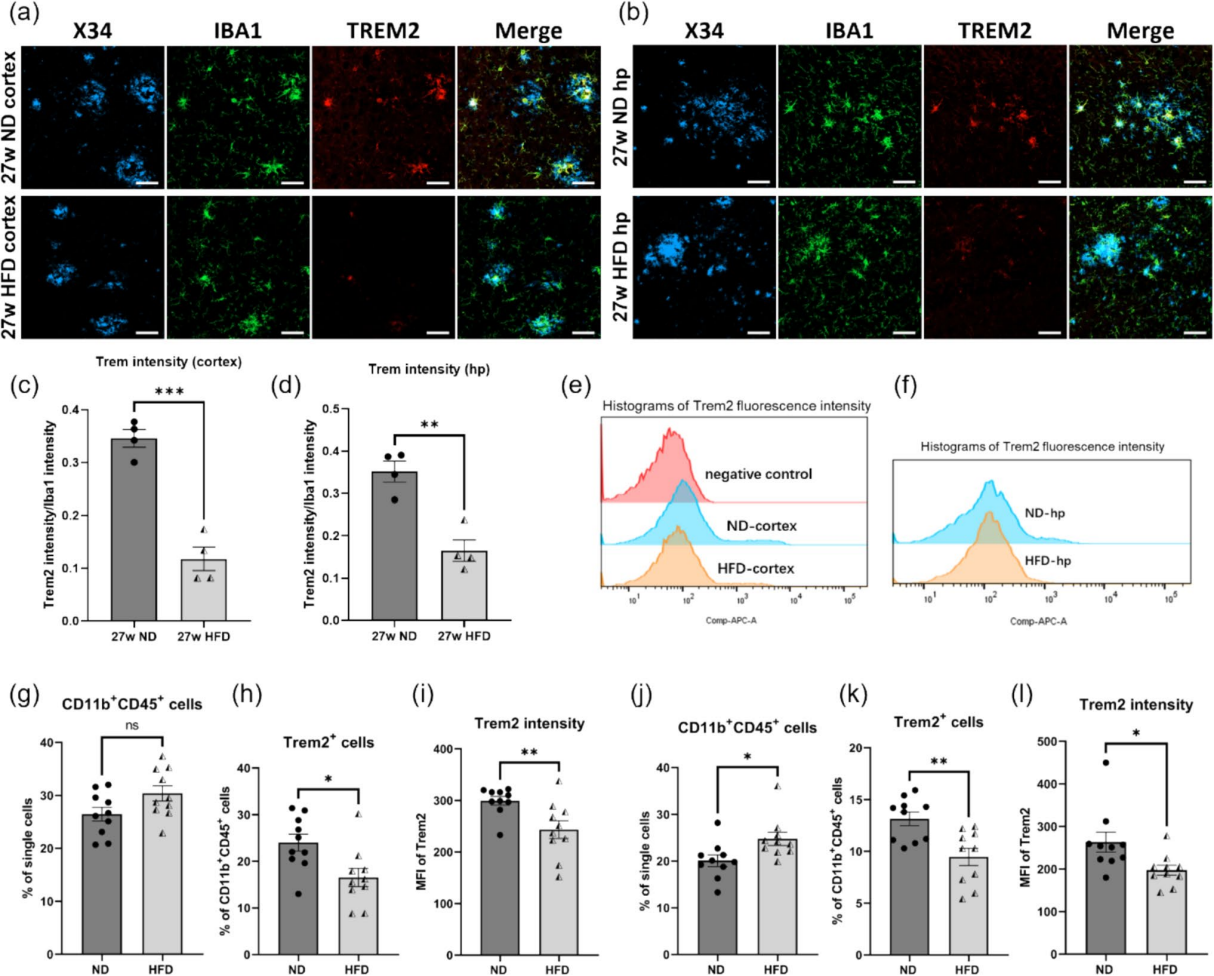
HFD feeding reduced TREM2 expression in microglia of *APP*^*NL–G–F*^ mice brain. (**a**-**b**) Representative images of somatosensory cortex and hippocampus from *APP*^*NL–G–F*^ mice fed an ND or an HFD for 27 weeks. Sections were stained with BAN50 (blue), IBA1 (green) and TREM2 (red). Scale bar = 50 μm. (**c**-**d**) Quantification of TREM2 immunofluorescence intensity of cortex and hippocampus from *APP*^*NL–G–F*^ mice. Each dot represents an analyzed mouse, and 35 images were taken from each mouse brain for cortical analysis and 10 images were taken from each mouse brain for hippocampal analysis (*n* = 4 mice per group). (**e**–**f**) Mean fluorescence intensity (MFI) graph of TREM2^+^ microglia isolated from cortex and hippocampus tissue samples from *APP*^*NL–G–F*^ mice fed with ND or HFD for 27 weeks, as determined by flow cytometry with gating on CD45 and CD11b. (**g**, **j**) Percentage of CD45^+^ CD11b^+^ cell population among all living single cells from the cortex and hippocampus tissue samples of *APP*^*NL–G–F*^ mice. (**h**, **k**) Percentage of TREM2^+^ cell population within CD45^+^ CD11b^+^ cell population from the cortex and hippocampus tissue samples of *APP*^*NL–G–F*^ mice. (**i**, **l**) MFI of TREM2 in the CD45^+^ CD11b^+^ population from the cortex and hippocampus tissue samples of *APP*.^*NL–G–F*^ mice. Values are average ± SEM. Statistical analyses were performed using an unpaired t-test (*p < 0.05, **p < 0.01; ns, not significant)

Since our results showed that OA treatment in vitro led to decreased TREM2 expression and impairment of lipid metabolism and phagocytic activity in MG6, we hypothesized that the impaired function of microglia in *APP*^*NL−G−F*^ mice induced by HFD was also related to the expression level of TREM2. We performed immunofluorescence staining of microglia and TREM2 in the cortex and hippocampus of *APP*^*NL−G−F*^ mice. Consistent with previous studies [48, 49], TREM2 was highly expressed in activated microglia near the Aβ plaques (Fig. 9a-b). Interestingly, the fluorescence intensity of TREM2 in microglia from HFD-fed *APP*^*NL−G−F*^ mice was significantly lower than that in microglia from the ND group (Fig. 9a-d). To confirm this result, we isolated microglia from the cerebral cortex and hippocampus of ND- and HFD-fed *APP*^*NL−G−F*^ mice and measured TREM2 expression levels using flow cytometry. HFD intake did not affect the proportion of CD45^+^ CD11b^+^ microglia in the cerebral cortex of *APP*^*NL−G−F*^ mice (Fig. 9g) but slightly increased the proportion of microglia in the hippocampus (Fig. 9j). Consistent with our expectations, in both cortex and hippocampus, the proportion of TREM2^+^ microglia was significantly reduced by HFD (Fig. 9h, k). Moreover, the mean fluorescence intensity (MFI) of TREM2 in isolated microglia was significantly reduced by HFD (Fig. 9e-f, 9i, 9 l).

As previously mentioned, TREM2 is a key regulator of microglial activation in AD brains, orchestrating the expression of several downstream genes associated with the full activation of microglia. To determine whether reduced TREM2 level in microglia of HFD-fed *APP*^*NL−G−F*^ mice impaired microglial activation, we assessed lipoprotein lipase (LPL), another DAM marker alongside TREM2, by immunohistochemistry. In the cortex, LPL was predominantly expressed in microglia surrounding Aβ plaques and was significantly lower in HFD-fed mice than in ND controls (Fig. S6a, b). While hippocampal microglia overall exhibited higher LPL levels, HFD still caused a marked reduction (Fig. S6a, c). The above results indicate that long-term HFD feeding downregulates the expression of TREM2 and LPL in microglia within the brains of *APP*^*NL−G−F*^ mice, which may explain the observed reduction of microglia near Aβ plaques, decreased microglial phagocytic activity, and the accumulation of LDs in the brains of HFD-fed *APP*^*NL−G−F*^ mice.

## Discussion

This study revealed that long-term HFD intake accelerated the progression of AD pathology in *APP*^*NL−G−F*^ mice brain. The underlying mechanism may involve HFD-induced accumulation of ChEs in the brain, leading to dysregulated lipid metabolism in microglia and excessive LD burden. Consequently, microglial recruitment to Aβ plaques and phagocytic function were impaired, ultimately reducing Aβ clearance.

Prospective studies have demonstrated that obesity (BMI > 30) during midlife significantly increases an individual's risk of developing dementia in their later life [50, 51]. To compare Aβ pathology progression between HFD and ND-fed *APP*^*NL−G−F*^ mice, we started HFD feeding at 8 weeks, aligning with the onset of Aβ plaque deposition in the cortex and hippocampus of *APP*^*NL−G−F*^ mice [15, 24]. After 17 weeks (at 25 weeks of age), the HFD group's average body weight was 1.29-fold that of the ND group; after 27 weeks (at 35 weeks of age), this ratio increased to 1.42-fold, exceeding the BMI ratio observed between obese individuals (BMI = 30) and those with a normal BMI of 22—and their serum total cholesterol had doubled relative to the ND group. Given that Aβ plaque deposition in *APP*^*NL−G−F*^ mice approaches saturation after 9 months of age, our study was conducted during the critical phase of plaque accumulation, thereby facilitating the investigation of the effects of HFD intake on pathological progression. In brief, our study emulated the impact of HFD-induced obesity on the progression of AD in human subjects.

Lipids are important components of the CNS, crucial for membrane formation and intracellular signaling. Their metabolism also plays a pivotal role in regulating microglial activation, function, and immune responses in neurodegenerative diseases [52]. A previous study detected elevated levels of diacylglycerol (DG) and sphingolipids in the prefrontal cortex of post-mortem AD patients and PS1-APP double transgenic AD mice. Additionally, increased levels of ganglioside GM3 and ChE were observed in their entorhinal cortex region [53]. Our lipidomic analysis results also indicate that in the *APP*^*NL−G−F*^ mouse cerebral cortex tissue, sphingolipids, including SM, Cer, HexCer, SPH and GM, were significantly elevated compared to WT mice. Research has elucidated that dysregulation of sphingolipid metabolism may lead to increased accumulation of Aβ, concomitantly instigating oxidative stress and apoptosis [54]. Study using matrix-assisted laser desorption/ionization imaging mass spectrometry (MALDI-IMS) also revealed the accumulation of gangliosides and ceramides within Aβ plaques, suggesting a close association between sphingolipids and Aβ metabolism [55]. However, there is insufficient evidence regarding the impact of sphingolipids on glial cells in the vicinity of Aβ plaques in AD brains. Furthermore, our two-factor analysis revealed that among the sphingolipid species, only SM exhibited an increase in response to both *APP*^*NL−G−F*^ gene knock-in and HFD intake, and this increase was marginal, with no statistically significant differences observed between any groups. These findings suggest that sphingolipids may not play a pivotal role in the HFD-induced exacerbation of plaque deposition in the *APP*^*NL−G−F*^ mice.

Importantly, both *APP*^*NL−G−F*^ gene knock-in and HFD individually elicited substantial increases in ChE levels in the cerebral cortex, and, they exhibited statistically significant interaction between the two factors (Fig. 5c), implying that the pathological state of AD may inherently augment the sensitivity of ChE metabolism to dietary factors, or conversely, that an HFD may exacerbate the abnormal accumulation of ChE triggered by *APP*^*NL−G−F*^ knock-in. Approximately 23% of the body's cholesterol is found in the CNS, where it plays a key role as a major component of myelin and cell membranes, supporting neuronal signaling, plasticity, and synaptic function [56]. Although nearly all cerebral cholesterol exists in its free form, a minute surplus undergoes esterification and is sequestered as ChE within neutral LDs. Especially, this esterification process is abnormally upregulated under pathological conditions [57]. In addition to ChE, another major lipid component of LD is TG. Our results indicate that although LDs in cortical microglia were significantly increased in the HFD group, the proportion of TG in the cerebral cortex remained unchanged in response to either HFD or AD pathology; therefore, we hypothesized that the increased LDs in the cortical microglia of HFD-fed *APP*^*NL−G−F*^ mice may predominantly contain a high content of ChE. In the brains of 5xFAD model mice, LDs were found to accumulate predominantly within microglia surrounding Aβ plaques, indicative of aberrant lipid metabolism in plaque-associated cells [58]. Moreover, long-term consumption of an HFD under physiological conditions results in LD accumulation and phenotypic alterations in hippocampal microglia, subsequently inducing behavioral abnormalities in mice [59]. In the cortex of *APP*^*NL−G−F*^ mice fed an ND, we also observed LD aggregation in activated microglia adjacent to plaques; notably, LD accumulation was markedly increased in the HFD group, suggesting that HFD further exacerbated the Aβ pathology-induced dysregulation of microglial lipid metabolism.

Previous reports have shown that microglia overloaded with LD exhibit decreased phagocytic ability, highlighting the relationship between lipid metabolic impairment and microglial function. LD-accumulating microglia have been observed in the hippocampi of aged mice, displaying a phenotype characterized by increased inflammation and reduced phagocytic activity [44]. Moreover, deletion of circadian clock protein REV-ERBα also enhanced inflammatory signaling, disrupted lipid metabolism, and induced LD accumulation in male microglia [60]. Conversely, reducing the burden of LDs on microglia by enhancing cholesterol efflux can reverse microglial dysfunction [61]. Although the mechanisms by which microglia accumulate LDs may differ under various pathological conditions or external stimuli, a common feature is that excessive LDs accumulation represents an impairment in lipid efflux, potentially altering the microglial phenotype. Our in vitro experiments found that OA—the most abundant FA in an HFD—specifically induced the accumulation of LDs in MG6 microglia and attenuated their ability to phagocytose Aβ peptides in a time-dependent manner. Because Sandoz 58–035 inhibits ChE synthesis via competitive binding to the ACAT1 active site without affecting TG formation, MG6 microglia co-treated with Sandoz 58–035 and OA still display LD accumulation; however, compared with cells treated with OA alone, they exhibit a marked reduction in LD burden and a restoration of Aβ-phagocytic activity. It is noteworthy that OA also profoundly disrupted their gene expression profiles. Our GOBP and GSEA results revealed that OA treatment downregulated a series of lipid metabolism-related pathways (including those involved in cholesterol homeostasis and efflux) and affected microglial immune defense responses and phagocytic/endocytic pathways, driving the cells toward a phenotype characterized by diminished immunosurveillance and responsiveness. Overall, these findings suggest that a high extracellular FA environment recapitulates the effects of prolonged HFD exposure on the CNS microenvironment, inducing shifts in microglial phenotypes and underscoring the link between lipid metabolism and immune cell function.

In addition, GSEA results also indicated that both the cholesterol efflux gene set and the phagocytosis/endocytosis gene set, which were downregulated following OA treatment, share several common leading-edge genes: *Abca1*, *Abca7*, *Apoa1*, *Apoa2*, *Scarb1*, and *Trem2*. In microglia, ABCA1 plays a key role in promoting the removal of excess intracellular cholesterol. Moreover, SCARB1 binds with APOA1 and APOA2, which are the main protein components of high-density lipoprotein (HDL), selectively mediating the uptake of ChEs from the core of HDL. Additionally, the phagocytic activity of microglia is closely associated with the composition and fluidity of their plasma membrane; thus, the downregulation of these genes may impair microglial phagocytosis by altering membrane microdomains and disrupting downstream signal transduction. Beyond mediating microglial phagocytosis of Aβ and facilitating the conversion of plaque-resident microglia into the DAM subtype in the AD brain [18], previous studies also reported that *Trem2* acts as a critical metabolic regulator that links lipid metabolism with effective microglial phagocytosis by maintaining the expression of key cholesterol-handling genes such as *Abca1* and *Abca7* under phagocytic challenges [62] [63]. Our study confirmed that both long-term HFD feeding and OA treatment significantly reduced TREM2 expression in microglia, suggesting that a high-fat environment may disrupt the balanced transport of cholesterol between the intracellular and extracellular compartments through a TREM2-mediated mechanism in microglia, ultimately downregulating a range of microglial protective functions. Further, *Lpl*, one of the specific genes of DAM, is markedly upregulated in plaque-associated microglia during late-stage AD [18]. Elevated LPL expression not only enhances FA metabolism but also boosts microglial phagocytic clearance of Aβ and lipid debris [64, 65]. Our study demonstrated a significant reduction of LPL levels in microglia from the HFD-fed *APP*^*NL−G−F*^ mice brains, indicating that HFD intake impeded the transition of microglia toward the fully activated DAM subtype.

Our study still has certain limitations. First, there are inherent differences in lipid metabolism between mice and humans. For example, HFD feeding does not significantly elevate mice serum TG levels (Fig. 1e) as previously reported [66], potentially due to the lack of cholesteryl ester transfer protein (CETP) activity in mice [67]. This deficiency impairs the conversion of very-low-density lipoprotein (VLDL) to low-density lipoprotein (LDL), leading to accumulation of VLDL remnants that are more efficiently cleared by the liver, thereby attenuating plasma TG level. Given the paucity of data on lipid composition in the brain tissue of obese individuals, the potential influence of TG on the human CNS cannot be excluded. Second, the relationship between lipid metabolism in peripheral tissues (including blood) and the CNS has not been clearly elucidated. An increase in ChE induced by HFD feeding was observed in both serum (Fig. 1e) and brain through lipidomic analysis (Fig. 5c), however, the mechanism underlying the elevation of ChE in the brain remains unclear. Nevertheless, our study indicate that the HFD induced increase in brain ChE following long-term feeding may contribute to the exacerbation of AD pathology, potentially through dysregulation of microglial function.

In conclusion, we demonstrated that long-term HFD intake exacerbates AD pathology and increases ChE levels in the brain of the *APP*^*NL−G−F*^ mouse model. Moreover, HFD-fed *APP*^*NL−G−F*^ mice exhibited impaired microglial responses, including reduced migration toward Aβ plaques, decreased Aβ uptake, and increased LD accumulation within microglia. These findings suggest that altered microglial ChE metabolism may contribute to these functional impairments. Elucidating the mechanisms underlying ChE accumulation in the brain and its impact on microglial function may aid in the development of novel therapeutic strategies for AD.

## Supplementary Information

Below is the link to the electronic supplementary material.

Supplementary file1 (DOCX 4597 KB)

## Data Availability

No datasets were generated or analysed during the current study.
