# Supplementary material for: High-Fat Diet-Induced Excessive Accumulation of Cerebral Cholesterol Esters and Microglial Dysfunction Exacerbate Alzheimer's Disease Pathology in APPNL−G−F mice
Source: Mol Neurobiol. 2025 May 17;62(9):12231–51. doi: 10.1007/s12035-025-05052-8 (PMC12367945; doi:10.1007/s12035-025-05052-8)
Supplement: Supplementary file 1 — Supplementary file1 (DOCX 4597 KB) [file 12035_2025_5052_MOESM1_ESM.docx]

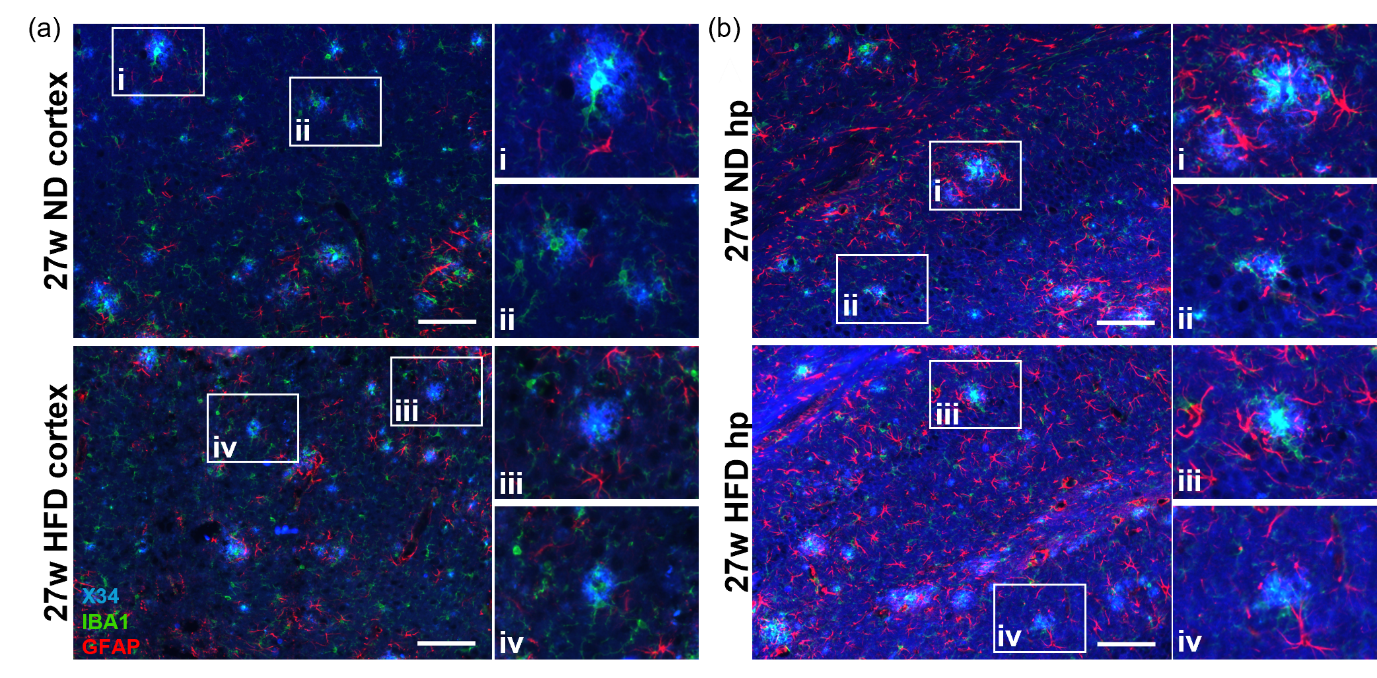


**Fig. S1** Activated microglia and reactive astrocytes accumulated around Aβ plaques. (a-b) Representative images of cortex and hippocampus from *APP^NL-G-F^* mice fed with ND or HFD for 27 weeks. Aβ plaques, microglia, and astrocytes are labeled with X34 (blue), IBA1 (green) and GFAP (red) respectively. Scale bar = 100 μm. The right panels show magnified views of the regions indicated by the white boxes. (a) In the cortex, nearly all plaques are surrounded by microglia, whereas astrocytes accumulate only around larger plaques and do not closely contact smaller plaques. (b) In the hippocampus, almost all plaques are surrounded by microglia and astrocytes, with microglia exhibiting a tighter association with the plaques. Activated microglia are selectively distributed near the plaques, while astrocytes display a more uniform distribution in the hippocampus.

**
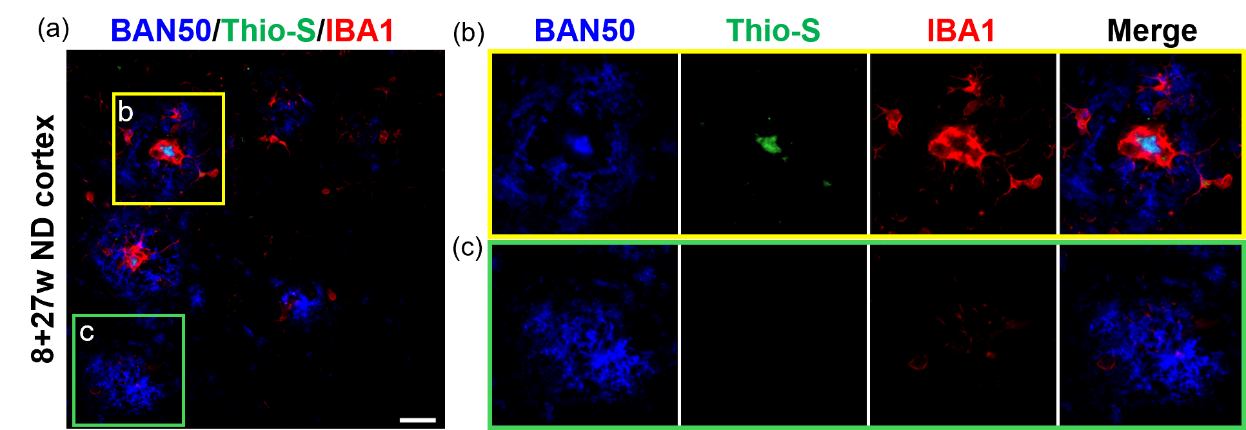
**

**Fig. S2** Formation of dense-core Aβ plaques requires the encapsulation and compaction of activated microglia. (a) Representative images of cortex from *APP^NL-G-F^* mice fed with ND stained with BAN50 (blue), Thioflavin-S (green) and IBA1 (red). Scale bar = 25 μm. (b-c) Higher magnification images of (a). The yellow box indicates a Thioflavin-S^+^ dense-core plaque enveloped by several activated microglia (b), whereas the green box indicates a Thioflavin-S^-^ diffuse plaque lack microglial encapsulation (c).

**
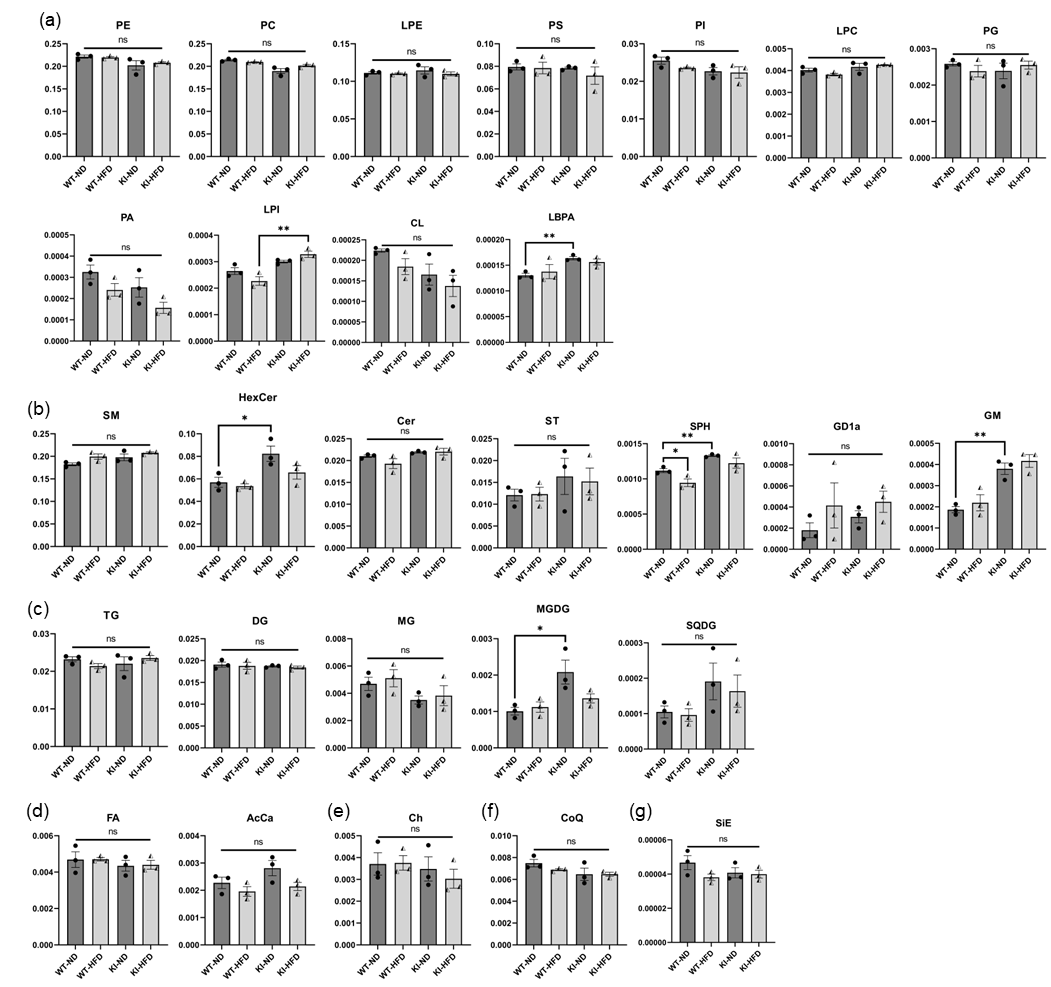
**

**Fig. S3** Proportions of lipid species in cortical tissue from WT and *APP^NL-G-F^* mice following 27 weeks of feeding with either an ND or an HFD. (a) Lipids within glycerophospholipids. (b) Lipids within sphingolipids. (c) Lipids within glycoglycerolipids. (d-g) Lipids within fatty acyls, sterol lipids and prenol lipids. Each dot represents an analyzed mouse brain. n = 3 mice per group. Values are average ± SEM. Statistical significance was determined using an unpaired t-test (**p* < 0.05, ***p* < 0.01; ns, not significant). The abbreviations and full names of the lipid species are as follows: PE: Phosphatidylethanolamine, PC: Phosphatidylcholine, LPE: Lysophosphatidylethanolamine, PS: Phosphatidylserine, PI: Phosphatidylinositol, LPC: Lysophosphatidylcholine, PG: Phosphatidylglycerol, PA: Phosphatidic acid, LPI: Lysophosphatidylinositol, CL: Cardiolipin, LBPA: Lysobisphosphatidic acid, SM: Sphingomyelin, HexCer: Glucosyl ceramide, Lactosyl ceramide and mainly Globotriaosyl ceramide, Cer: Ceramide, ST: Sulfatide, SPH: Sphingosine, GD1a: Disialotetrahexosylganglioside, GM: Ganglioside monosialo, TG: Triglyceride, DG: Diglyceride, MG: Monoglyceride, MGDG: Monogalactosyldiacylglycerol, SQDG: Sulfoquinovosylmonoacylglycerol, AcCa: Acyl Carnitine, Ch: Cholesterol, CoQ: Coenzyme Q, SiE: Sitosterol ester.

**
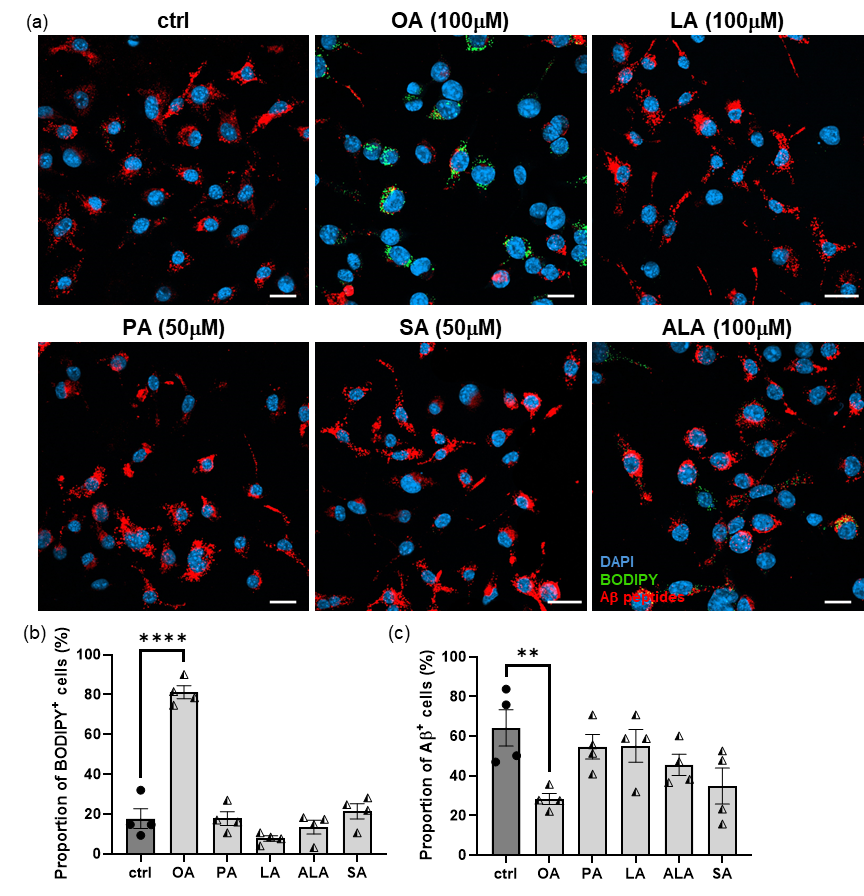
**

**Fig. S4** OA, instead of other FAs, induced LDs accumulation in MG6 and downregulated MG6 phagocytic activity. (a) Representative images of MG6 cells treated with 100 μM OA, 50 μM PA, 100 μM LA, 100 μM ALA or 50 μM SA for 24 hours, followed by incubation with 1μM of HiLyte™ Fluor 555-labeled Aβ_1-42_ for 3 hours, and subsequently stained with BODIPY and DAPI. The culture medium of the control group was not supplemented with FAs. Scale bars = 20 μm. (b) The proportion of BODIPY^+^ cells. Only OA treatment significantly induced LD accumulation among all FAs. (c) The proportion of cells that internalized Aβ. Only OA treatment significantly downregulated Aβ uptake by MG6. Each dot represents an individual experiment (n = 4). 8 images were taken and analyzed for each sample in each experiment. Values are average ± SEM. Statistical significance was determined using an unpaired t-test (***p* < 0.01, *****p* < 0.0001; no symbol indicates no statistically significant difference).

**
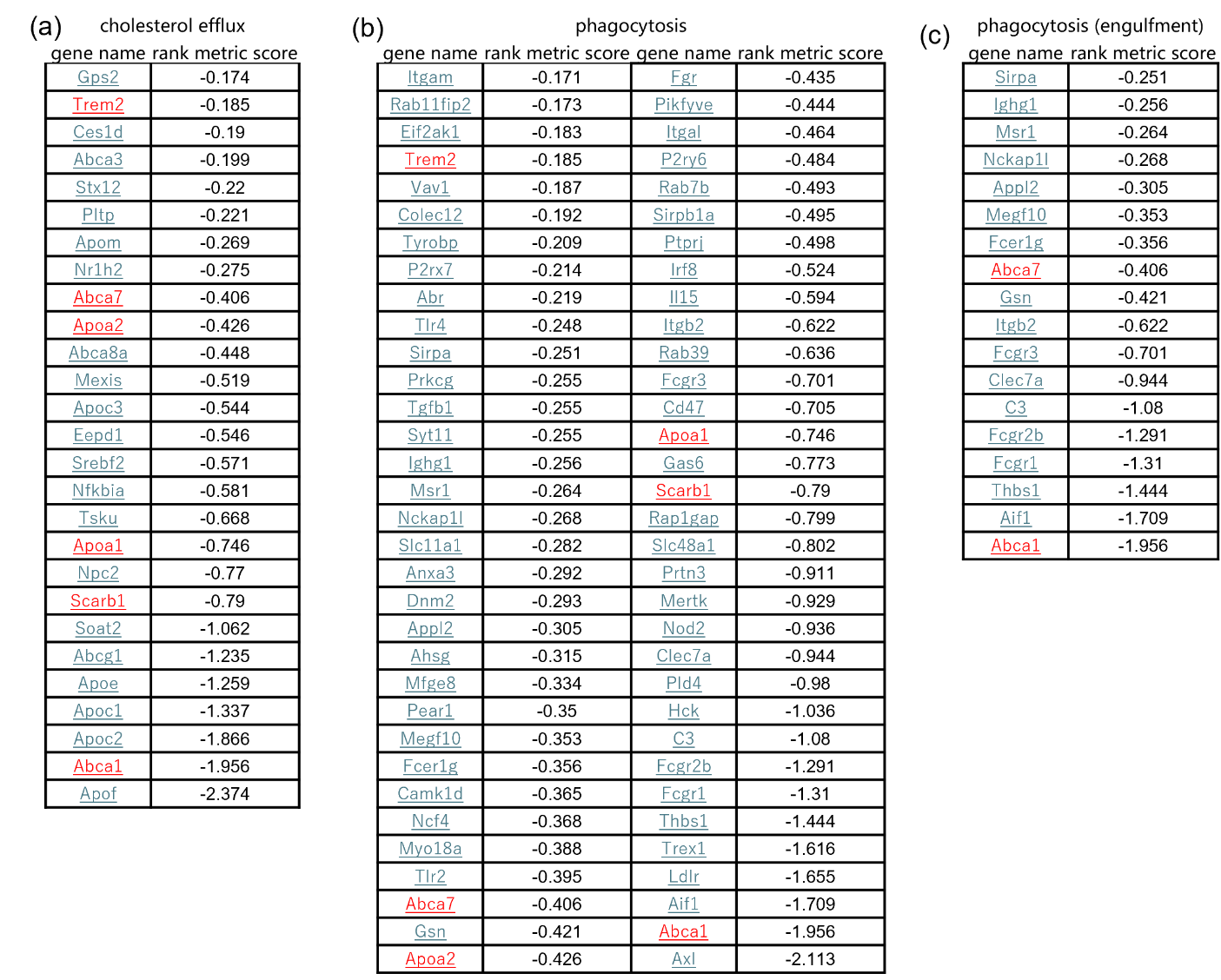
**

**Fig. S5** Leading-edge genes in cholesterol efflux, phagocytosis, and engulfment gene sets. (a-c) The table lists the leading-edge genes and their rank metric scores from the three gene sets that were significantly downregulated in MG6 microglial cells after 24 hours of OA treatment compared to the control MG6 cells. Genes marked in red are the leading-edge genes common to both cholesterol efflux and phagocytosis/ engulfment gene sets.


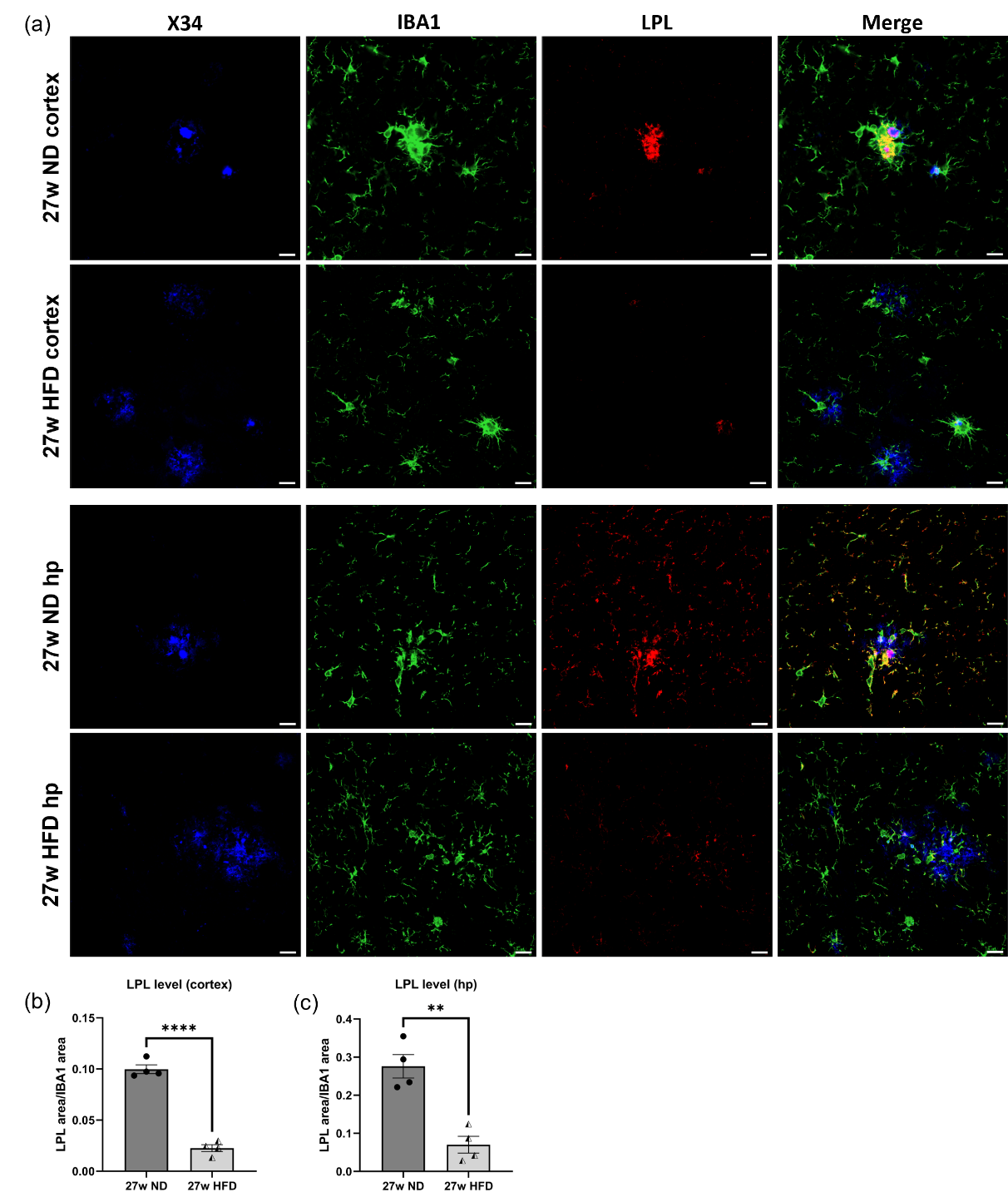


**Fig. S6** HFD reduced microglial LPL expression in the cortex and hippocampus of *APP^NL-G-F^* mice. (a) Representative images of somatosensory cortex and hippocampus from *APP^NL-G-F^* mice fed an ND or an HFD for 27 weeks. Sections were stained with X34 (blue), IBA1 (green) and LPL (red). Scale bar = 15 μm. (b-c) Quantitative analysis of LPL expression in microglia was performed by calculating the ratio of the overlapping area of LPL and IBA1 immunofluorescence to the total IBA1^+^ area. Each dot represents an analyzed mouse, and 16 images were taken from each mouse brain for cortical analysis and 8 images were taken from each mouse brain for hippocampal analysis (n = 4 mice per group). Values are average ± SEM. Statistical analyses were performed using an unpaired t-test (***p* < 0.01, **** *p* < 0.0001).
